# Supplementary material for: Microbial peptides activate tumour-infiltrating lymphocytes in glioblastoma
Source: Nature. 2023 May 17;617(7962):807–17. doi: 10.1038/s41586-023-06081-w (PMC10208956; doi:10.1038/s41586-023-06081-w)
Supplement: Supplementary file 1 — This file contains Supplementary Tables 1, 2, 9, 12, 13 and 16. [file 41586_2023_6081_MOESM1_ESM.pdf]

nature portfolio

https://doi.org/10.1038/s41586-023-06081-w

Supplementary information

Microbial peptides activate tumour-infiltrating lymphocytes in glioblastoma

In the format provided by the authors and unedited

Table of content - supplementary tables:

**Supplementary Table 1**	**Information about glioblastoma patients, whose tissue samples had been used for immunopeptidome analyses**
**Supplementary Table 2**	**HLA typing of glioblastoma patients**
**Supplementary Table 3**	**IPdBPs of glioblastoma patients**
**Supplementary Table 4**	**IPdBPs of control cohort**
**Supplementary Table 5**	**16S rRNA sequencing of primary and recurrent tumor tissues of patient 1635WI**
**Supplementary Table 6**	**16s rRNA sequencing of tumor samples from 9 additional glioblastoma patients**
**Supplementary Table 7**	**Comparison of bacterial species found via 16S rRNA sequencing with those found by immunopeptidomics**
**Supplementary Table 8**	**IPdBPs of primary and recurrent tumors (1635WI)**
**Supplementary Table 9**	**IPdBPs' fragmentation spectra (1635WI)**
**Supplementary Table 10**	**All synthetized peptides from ps-SCL tested with TCC88**
**Supplementary Table 11**	**Autoantigen peptide pools tested with TCC88**
**Supplementary Table 12**	**List of TCRBV antibodies and their corresponding IMGT annotation**
**Supplementary Table 13**	**Peptides used in vaccine- and bacteria/microbiota pools**
**Supplementary Table 14**	**Gene expression of the recurrent glioblastoma compared with glioblastomas in the TCGA Database**
**Supplementary Table 15**	**Comparison between bacterial peptides found via unbiased ps-SCL and IPdBPs**
**Supplementary Table 16**	**Comparison mass spectrometry spectra of synthetic peptides with IPdBPs**

Supplementary Table 1. Information about glioblastoma patients, whose tissue samples had been used for immunopeptidome analyses

ID	Gender	Age	Tumor type	IDH1 status/<br>MGMT status	Tissue/<br>cell line
1635WI	Male	55	Primary&<br>recurrent	WT<br>–	+<br>-
P ZH613	Male	74	Primary	WT<br>–	+<br>-
P ZH616	Female	86	Primary	WT<br>–	+<br>-
P ZH617	Female	74	Primary	WT<br>+	+<br>-
P ZH631	Male	60	Primary	WT<br>+	+<br>-
P ZH645	Male	66	Primary	WT<br>–	+<br>-
P ZH654	Male	72	Primary	WT<br>+	+<br>-
P ZH678	Male	66	Primary	WT<br>–	+<br>-
P ZH681	Male	78	Primary	WT<br>+	+<br>+
P ZH720	Male	70	Primary	WT<br>–	+<br>+
P ZH750	Female	60	Primary	WT<br>–	+<br>+
P ZH757	Male	70	Primary	WT<br>–	+<br>+
P ZH761	Male	80	Primary	WT<br>+	+<br>-
P ZH791	Male	63	Primary	WT<br>–	+<br>-
P ZH802	Female	70	Primary	WT<br>–	+<br>+
P ZH810	Female	53	Primary	WT<br>+	+<br>-
P ZH829	Female	70	Primary	WT<br>–	+<br>-
R ZH753	Female	59	Recurrent	WT<br>+	+<br>+
R ZH784	Male	63	Recurrent	WT<br>+	+<br>-
GBM-40	Male	46	Recurrent	WT<br>–	-<br>+

Supplementary Table 2. HLA typing of glioblastoma patients

ID	HLA-I	HLA-II
1635WI	A*03:02;A*26:01;B*08:01;B*51:01	DRB1*03:01;DRB1*04:02;DRB3*02:02;DRB4*01:01; DQA1*03:01;DQA1*05:01;DQB1*02:01;DQB1*03:02
P ZH613	A*02:05;A*31:01;B*51:01;B*58:01;C*05:01;C*07:01	DRB1*13:01;DRB1*13:02;DRB3*02:02;DRB3*03:01; DQB1*06:09;DQB1*06:03;DQA1*01:03;DQA1*01:02
P ZH616	A*02:01;A*29:02;B*07:02;B*44:02;C*05:01;C*07:02	DRB1*12:01;DRB1*15:01;DRB3*02:02;DRB5*01:01; DQB1*03:01;DQB1*06:02;DQA1*01:02;DQA1*05:01
P ZH617	A*01:01;A*02:01;B*08:01;B*44:02;C*05:01;C*07:01	DRB1*03:01;DRB1*03:01;DRB3*01:01;DRB3*01:01; DQB1*02:01;DQB1*02:01;DQA1*05:01;DQA1*05:01
P ZH631	A*11:01;A*32:01;B*44:02;B*51:01;C*03:03;C*07:04	DRB1*01:01;DRB1*08:01;DQB1*04:02;DQB1*05:01; DQA1*01:01;DQA1*04:01
P ZH645	A*01:01;A*02:01;B*49:01;B*51:01;C*01:02;C*07:01	DRB1*07:01;DRB1*13:02;DRB3*03:01;DRB4*01:01; DQB1*02:01;DQB1*06:04;DQA1*01:02;DQA1*02:01
P ZH654	A*01:01;A*31:01;B*51:01;B*57:01;C*06:02;C*15:06	DRB1*04:04;DRB1*07:01;DRB4*01:03;DRB4*01:01; DQB1*03:03;DQB1*03:02;DQA1*02:01;DQA1*03:01
P ZH678	A*02:01;A*11:01;B*44:03;B*51:01;C*04:01;C*15:02	DRB1*11:01;DRB1*11:01;DRB3*02:02;DRB3*02:02; DQB1*03:01;DQB1*03:01;DQA1*05:01;DQA1*05:01
P ZH681	A*02:01;A*02:01;B*07:02;B*57:01;C*06:02;C*07:02	DRB1*07:01;DRB1*13:02;DRB3*03:01;DRB4*01:03; DQB1*03:03;DQB1*06:04;DQA1*01:02;DQA1*02:01
P ZH720	A*02:01;A*02:01;B*18:01;B*39:10;C*07:01;C*12:03	DRB1*13:01;DRB1*13:01;DRB3*01:01;DRB3*01:01; DQB1*06:03;DQB1*06:03;DQA1*01:03;DQA1*01:03
P ZH750	A*02:01;A*24:02;B*15:17;B*44:02;C*07:01;C*07:04	DRB1*11:01;DRB1*13:02;DRB3*02:02;DRB3*03:01; DQB1*03:01;DQB1*06:04;DQA1*01:02;DQA1*05:01
P ZH757	A*02:01;A*02:01;B*07:02;B*27:05;C*01:02;C*07:02	DRB1*08:01;DRB1*15:01;DRB5*01:01;DQB1*04:02; DQB1*06:02;DQA1*01:02;DQA1*04:01
P ZH761	A*02:01;A*03:01;B*44:02;B*51:26;C*01:02;C*16:01	DRB1*01:01;DRB1*12:01;DRB3*02:02;DQB1*03:01; DQB1*05:01;DQA1*01:01;DQA1*05:01
P ZH791	A*03:01;A*11:01;B*07:02;B*13:02;C*06:02;C*07:02	DRB1*11:01;DRB1*15:01;DRB3*02:02;DRB5*01:01; DQB1*03:01;DQB1*06:02;DQA1*01:02;DQA1*05:01
P ZH802	A*31:01;A*33:01;B*14:02;B*44:02;C*05:01;C*08:02	DRB1*04:04;DRB1*13:02;DRB3*03:01;DRB4*01:01; DQB1*03:02;DQB1*06:04:01;DQA1*01:02;DQA1*03:01
P ZH810	A*24:02;A*24:02;B*44:03;B*57:01;C*04:01;C*06:02	DRB1*07:01;DRB1*11:01;DRB3*02:02;DRB4*01:01; DQB1*03:03;DQB1*03:01;DQA1*02:01;DQA1*05:01
P ZH829	A*02:01;A*02:01;B*44:05;B*55:01;C*01:02;C*02:02	DRB1*13:01;DRB1*16:01;DRB3*02:02;DRB5*02:02; DQB1*05:02;DQB1*06:03;DQA1*01:03;DQA1*01:02
R ZH753	A*02:01;A*02:01;B*08:01;B*51:01;C*07:01;C*15:02	DRB1*01:01;DRB1*03:01;DRB3*01:01;DQB1*02:01; DQB1*05:01;DQA1*01:01;DQA1*05:01
R ZH784	A*01:01;A*02:01;B*07:02;B*08:01;C*07:01;C*07:02	DRB1*11:01;DRB1*15:01;DRB3*02:02;DRB5*01:01; DQB1*03:01;DQB1*06:02;DQA1*01:02;DQA1*05:01
GBM-40	A*01:01;A*68:01;B*44:02;B*57:01; C*06:02;C*07:04	DRB1*07:01;DRB1*11:01; DQB1*03:01;DQB1*03:03;DQA1*02:01;DQA1*05:05

Supplementary Table 9. IPdBPs' fragmentation spectra (1635WI)

Peptidome-Bac 1

ENLPVVLPL Mw (monoisotopic mass): 992.59

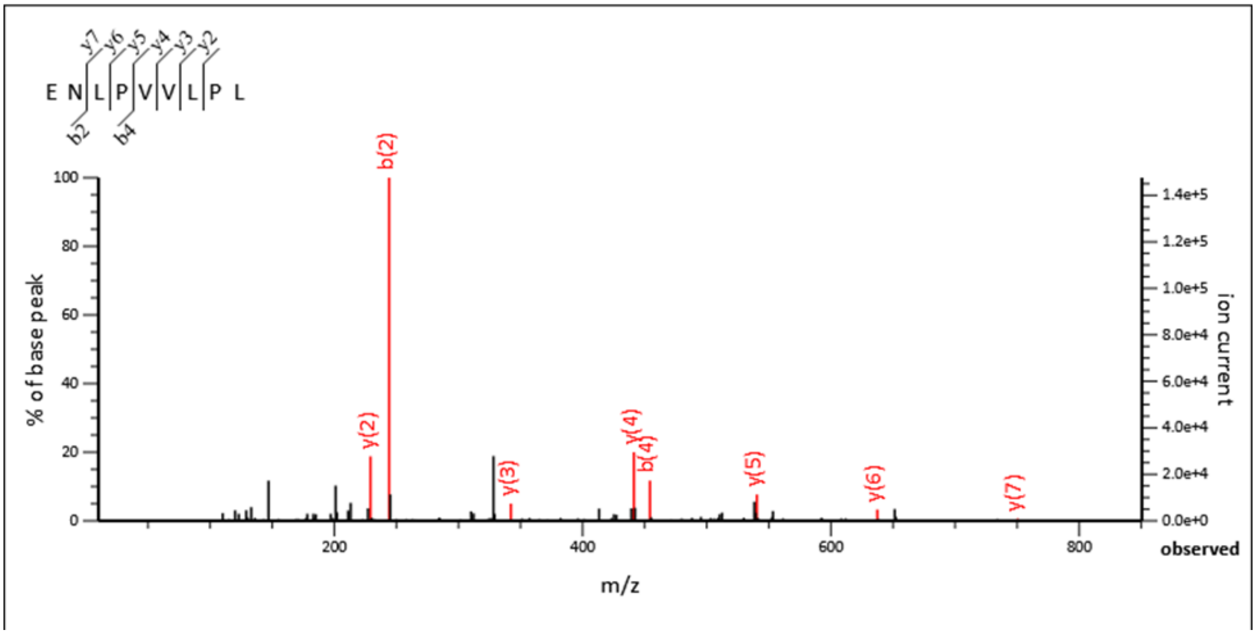

Peptidome-Bac 2

KVMLVDDVITAGTAIRE Mw (monoisotopic mass): 1829.99

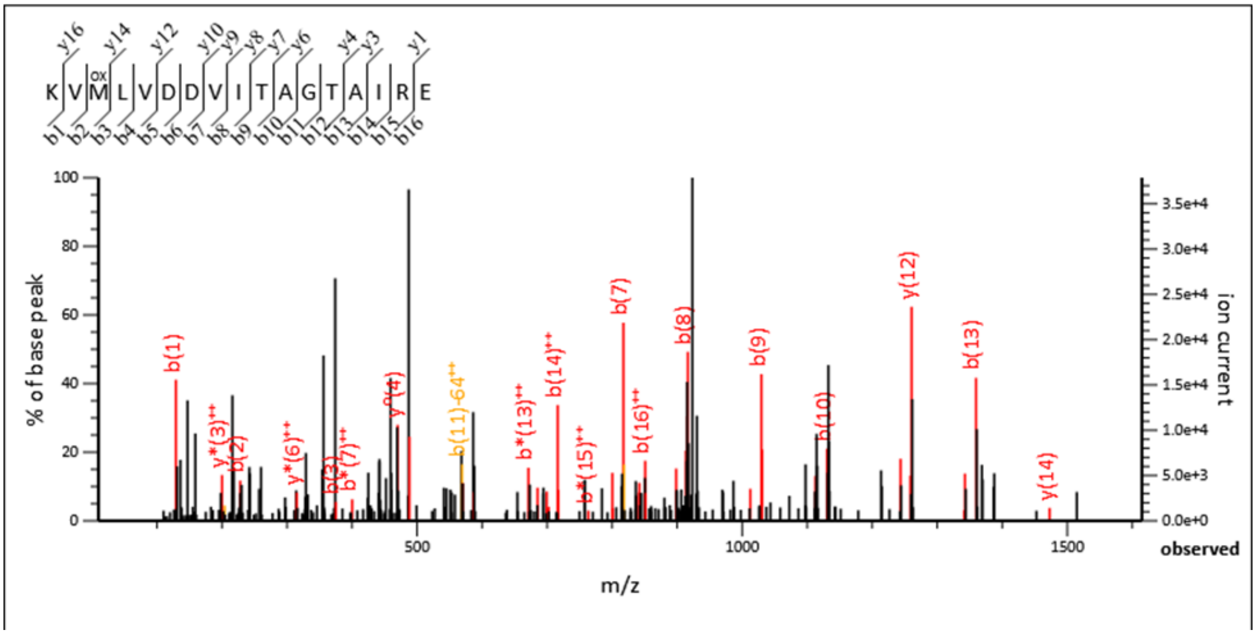

Peptidome-Bac 4

VEAVALQLKPLCKE Mw (monoisotopic mass): 1539.87

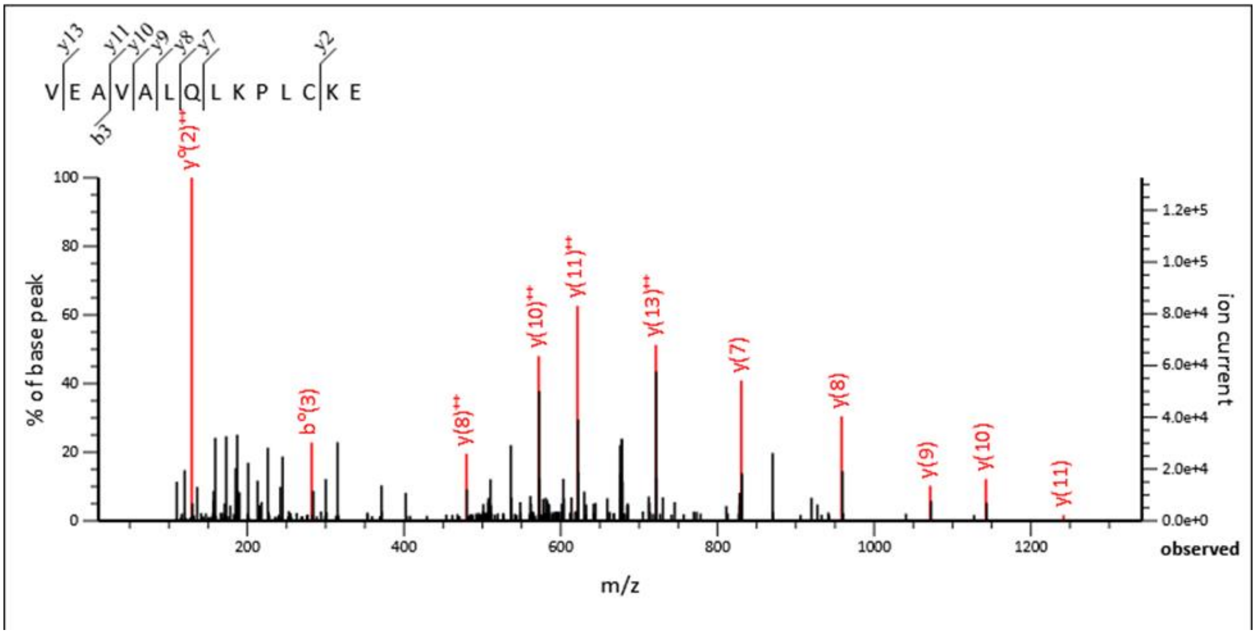

Peptidome-Bac 5

VFAIDRAGLVGEDGPT Mw (monoisotopic mass): 1615.82

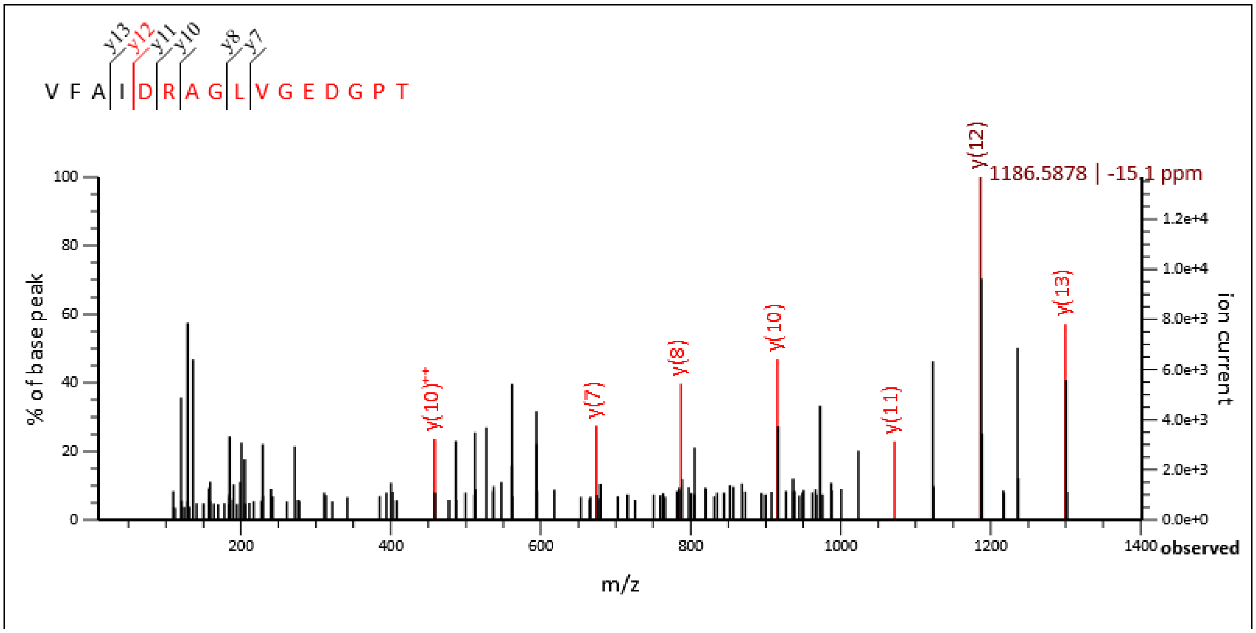

Peptidome-Bac 6

AALGGEMKNPEKL Mw (monoisotopic mass): 1356.71

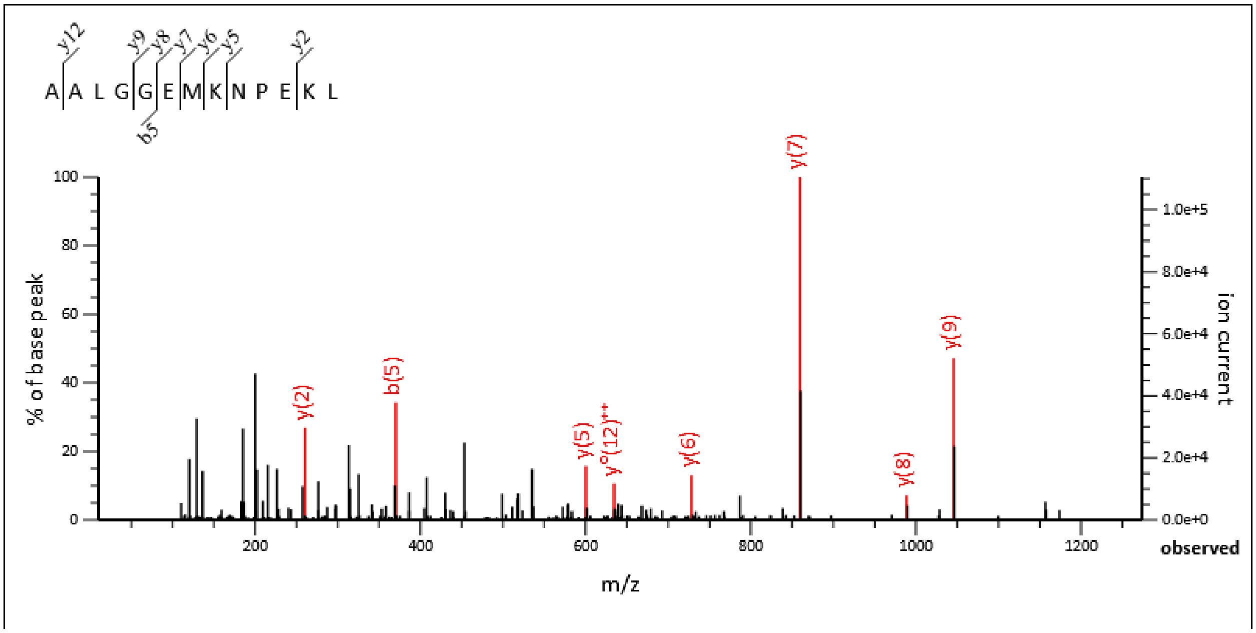

Peptidome-Bac 7

AGPVVAAAVILKEDC Mw (monoisotopic mass): 1454.78

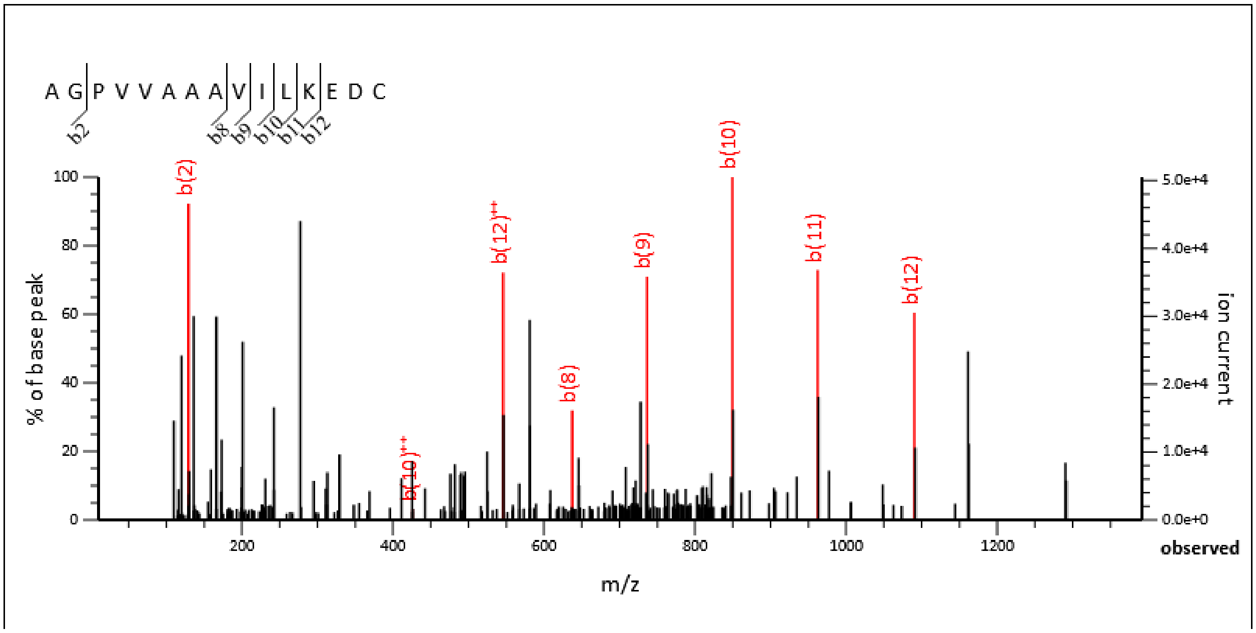

Peptidome- Bac 8

ALGQFMLDLHVNEH Mw (monoisotopic mass): 1622.79

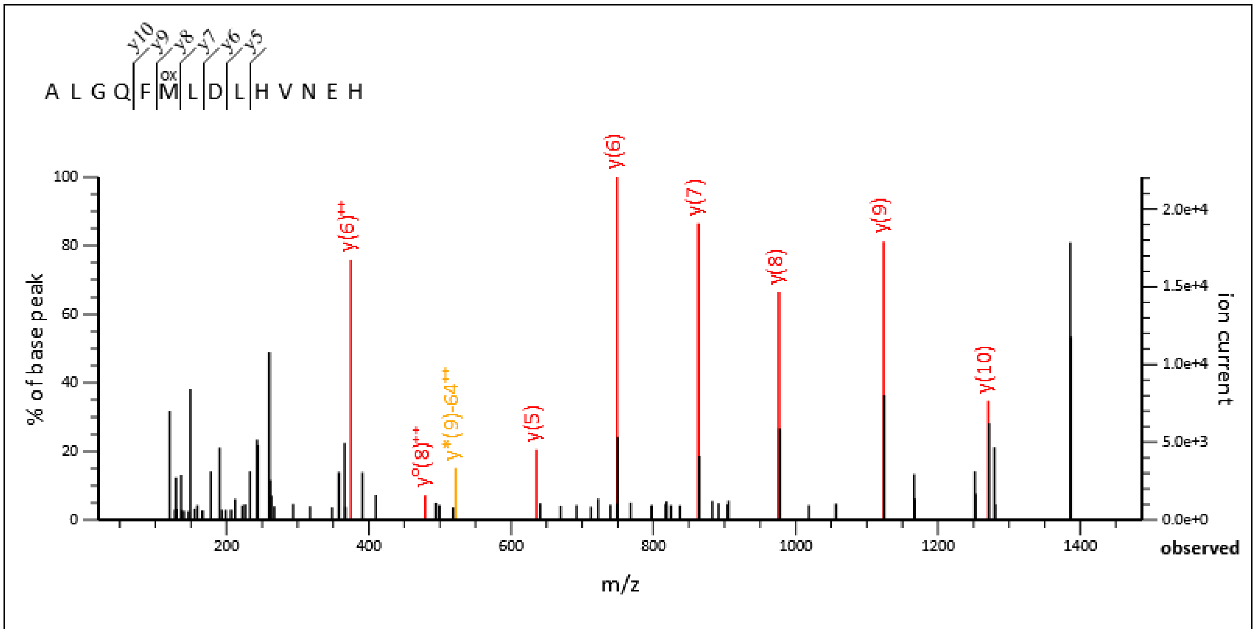

Peptidome-Bac 9

AVVTQPDK Mw (monoisotopic mass): 856.47

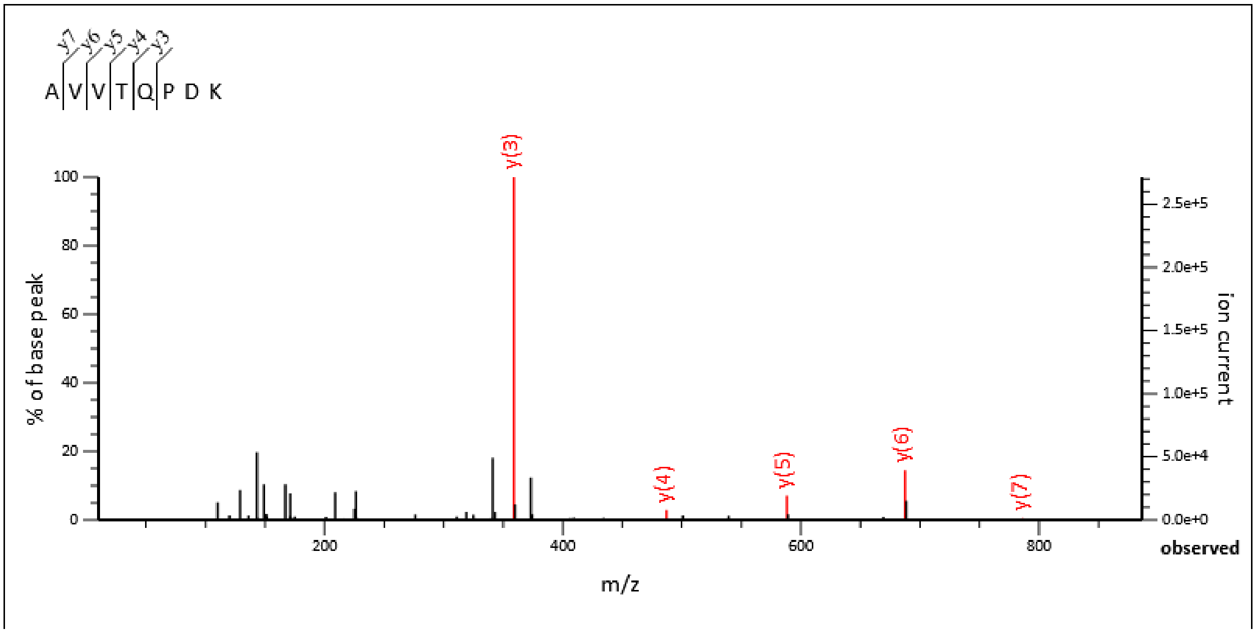

Peptidome-Bac 10

KEDMSVPLIKSIRK Mw (monoisotopic mass): 1642.94

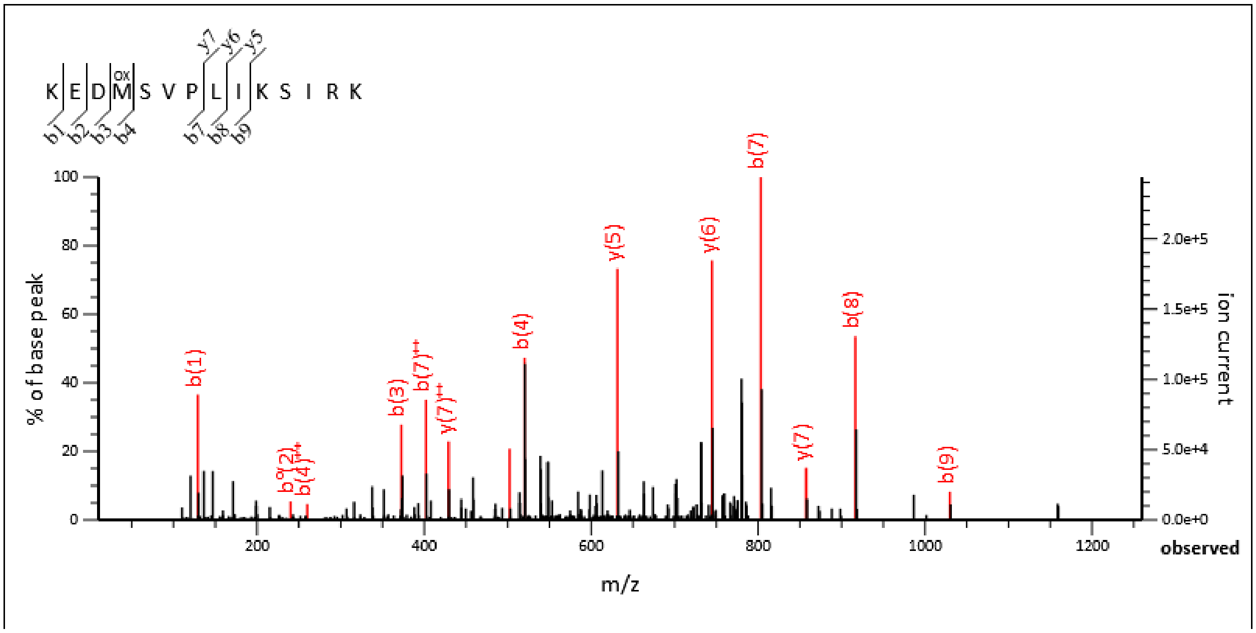

Peptidome-Bac 11

KNAGFWRTLFA Mw (monoisotopic mass): 1309.69

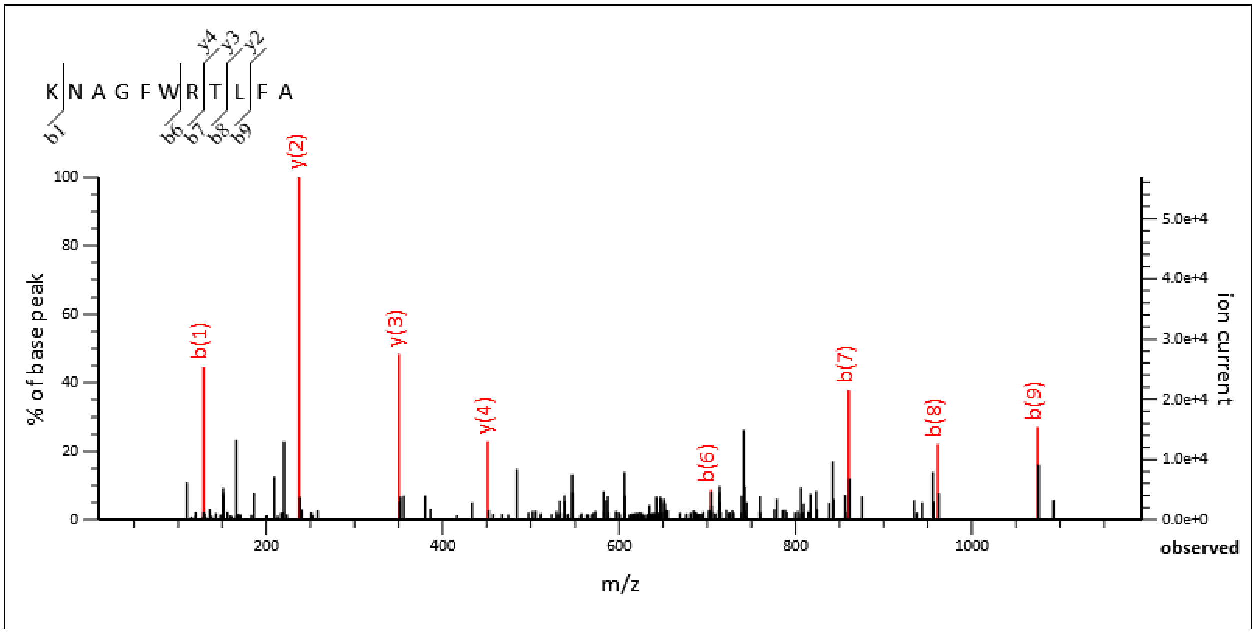

Peptidome-Bac 12

NLLDSMDDKVNLNGGAI Mw (monoisotopic mass): 1787.87

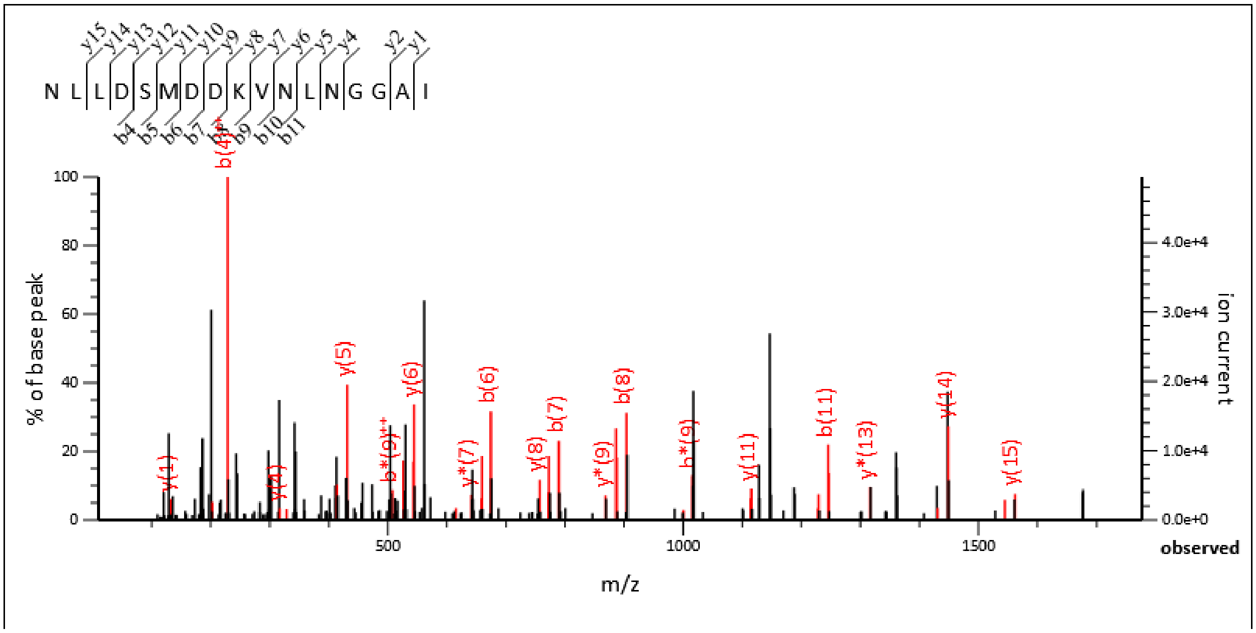

Peptidome-Bac 13

NLTVGAPMVAGAKVEAK Mw (monoisotopic mass): 1654.91

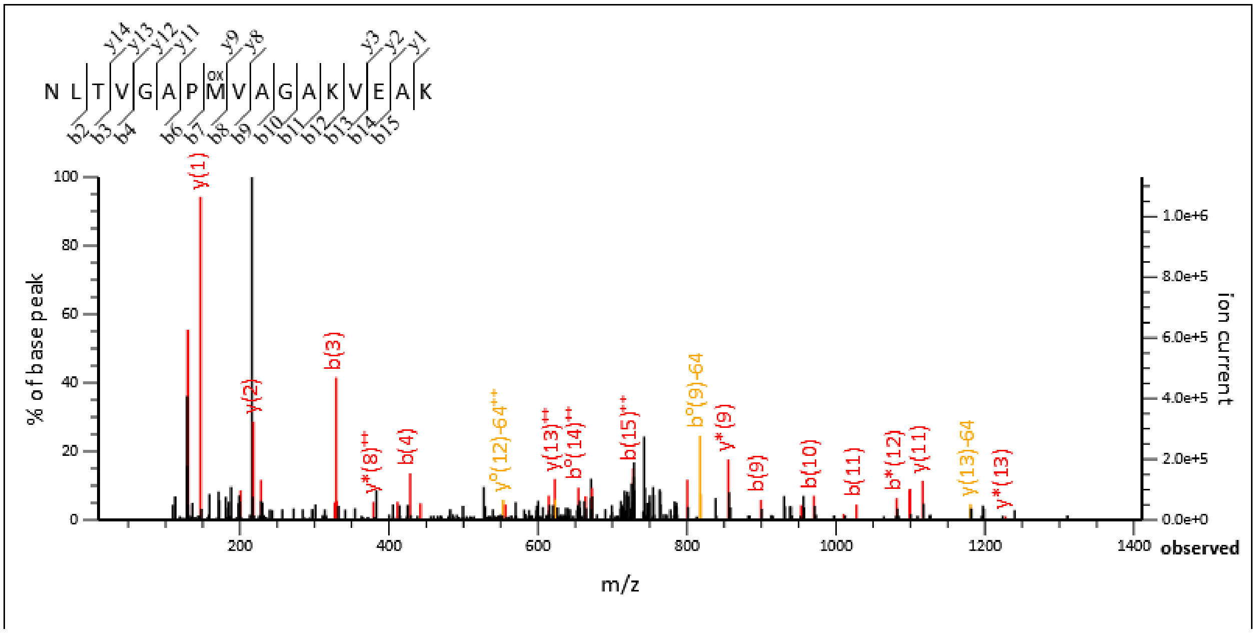

Peptidome-Bac 14

QALVTLEENAVIG Mw (monoisotopic mass): 1355.73

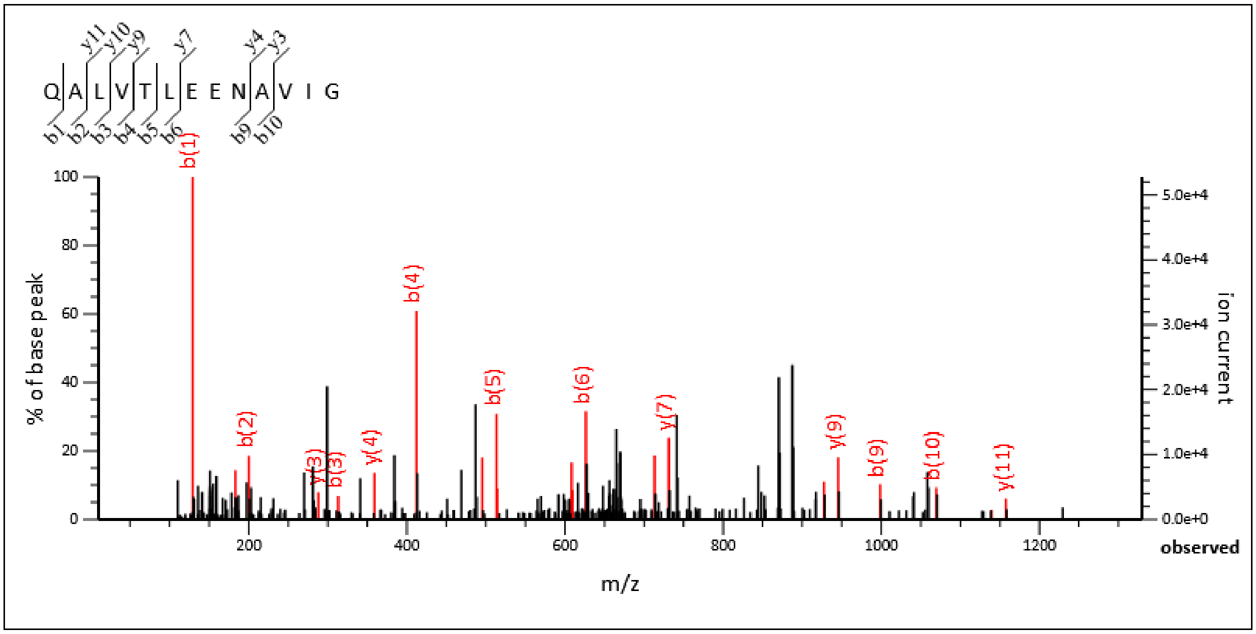

Peptidome-Bac 16

SNGDLDAIVVARAGLA Mw (monoisotopic mass): 1540.82

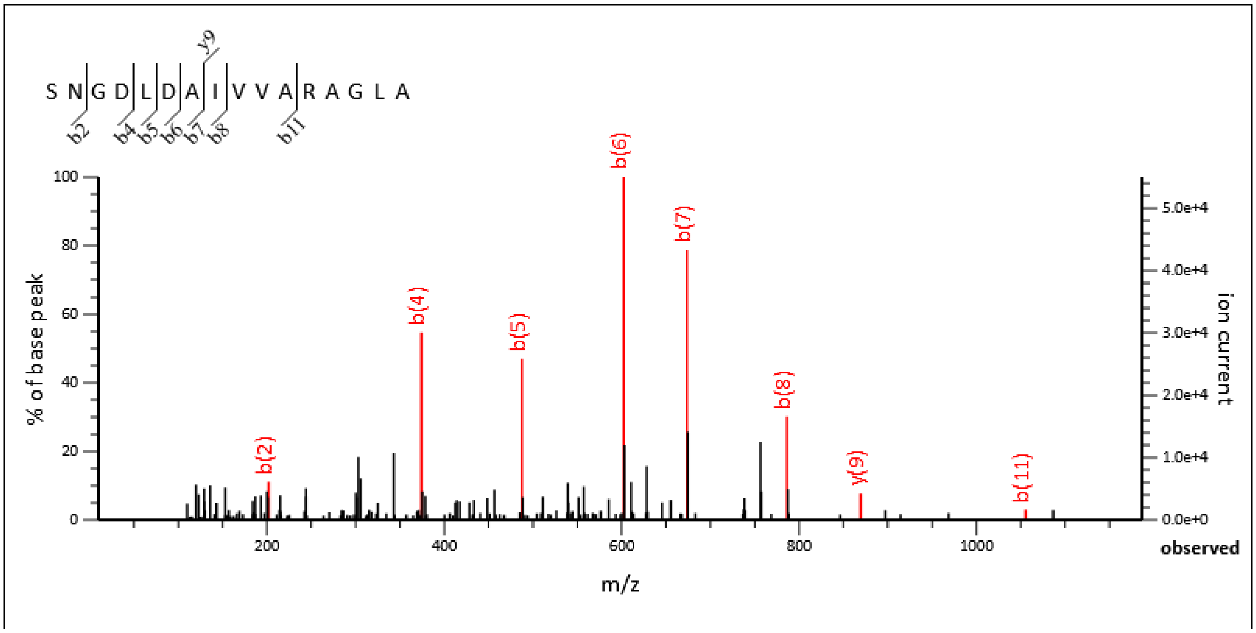

Peptidome-Bac 17

SNTPIIVDGKDVMPEVN Mw (monoisotopic mass): 1826.91

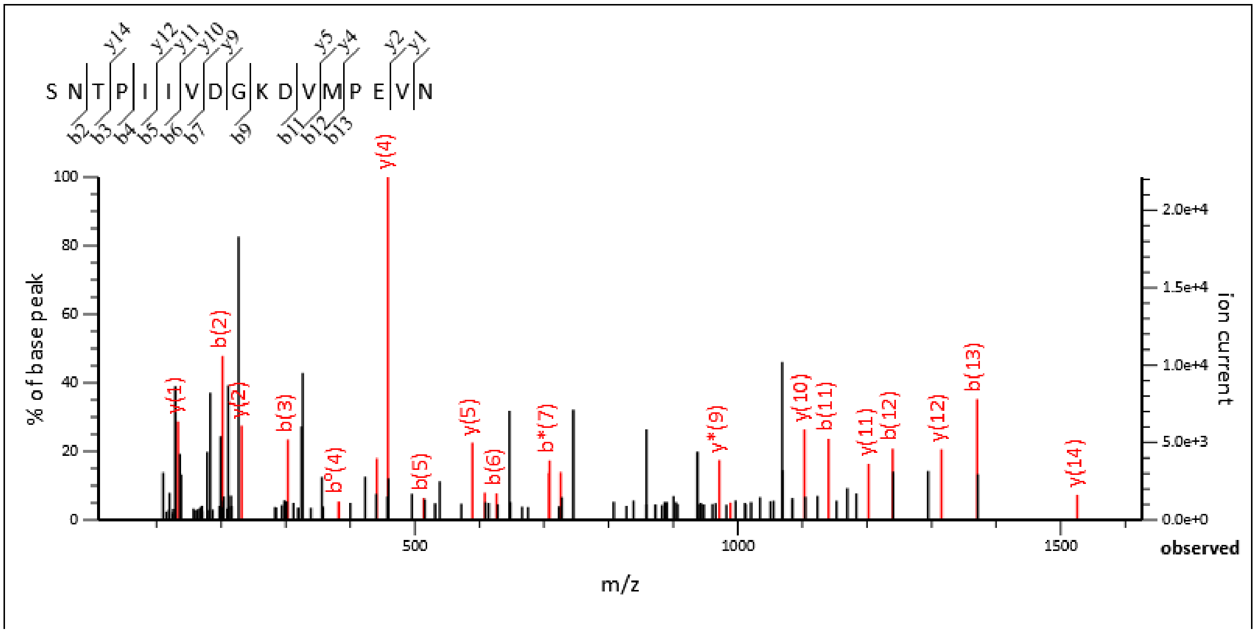

Peptidome-Bac 18

TPIIVDGKDVMPE Mw (monoisotopic mass): 1412.72

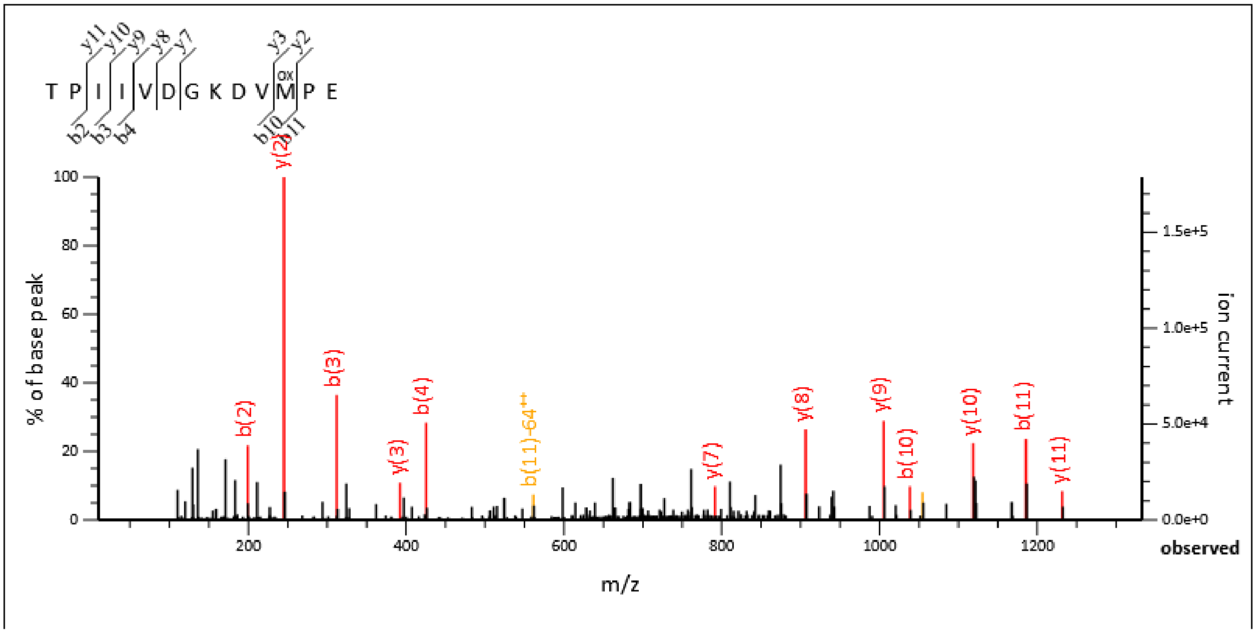

Peptidome-Bac 19

TPIIVDGKDVMPEVN Mw (monoisotopic mass): 1625.83

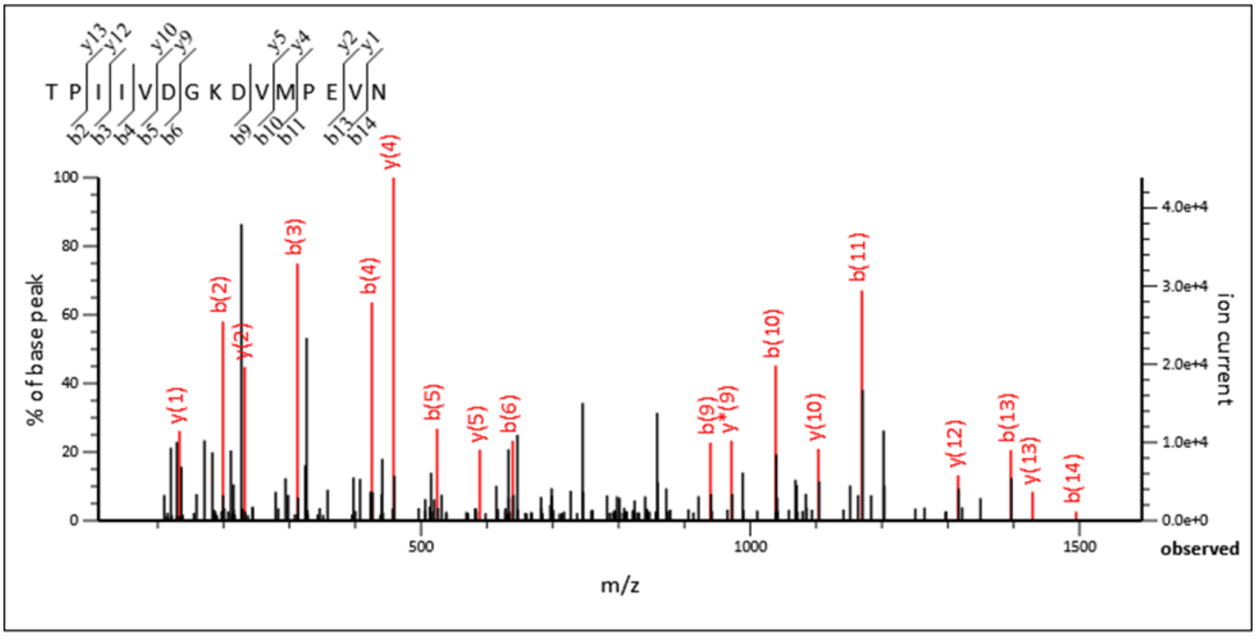

Peptidome-Bac 20

VTVLYGSQTGNAQGLAE Mw (monoisotopic mass): 1706.85

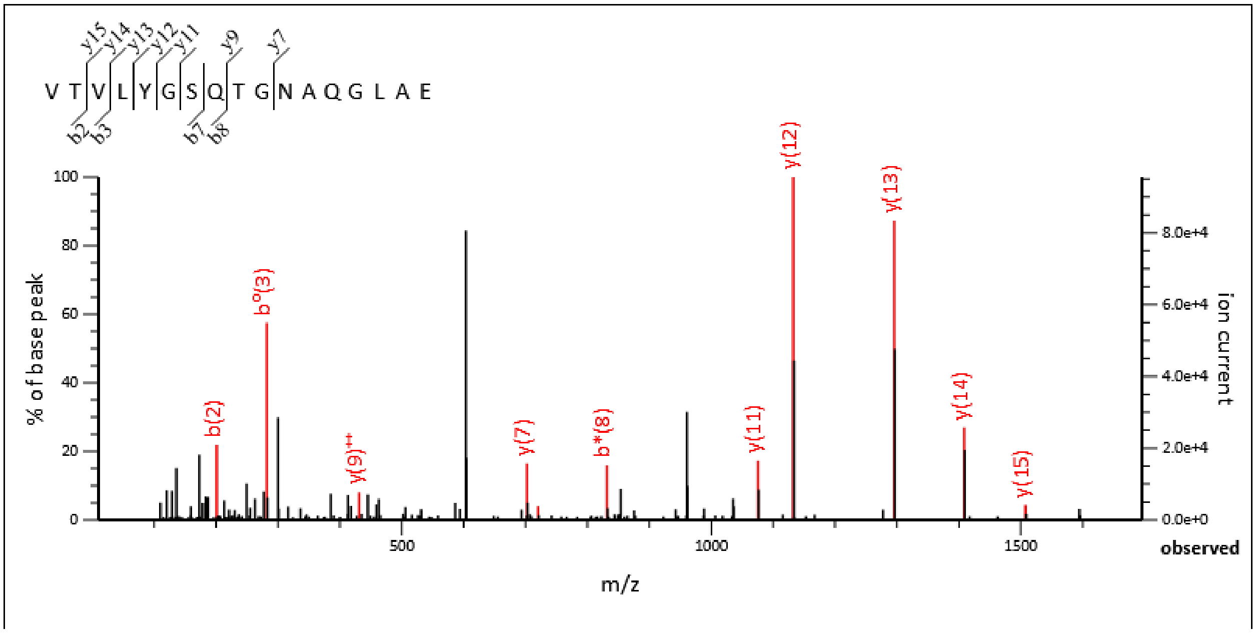

Peptidome-Bac 21

AAIAIGILLGH Mw (monoisotopic mass): 1047.64

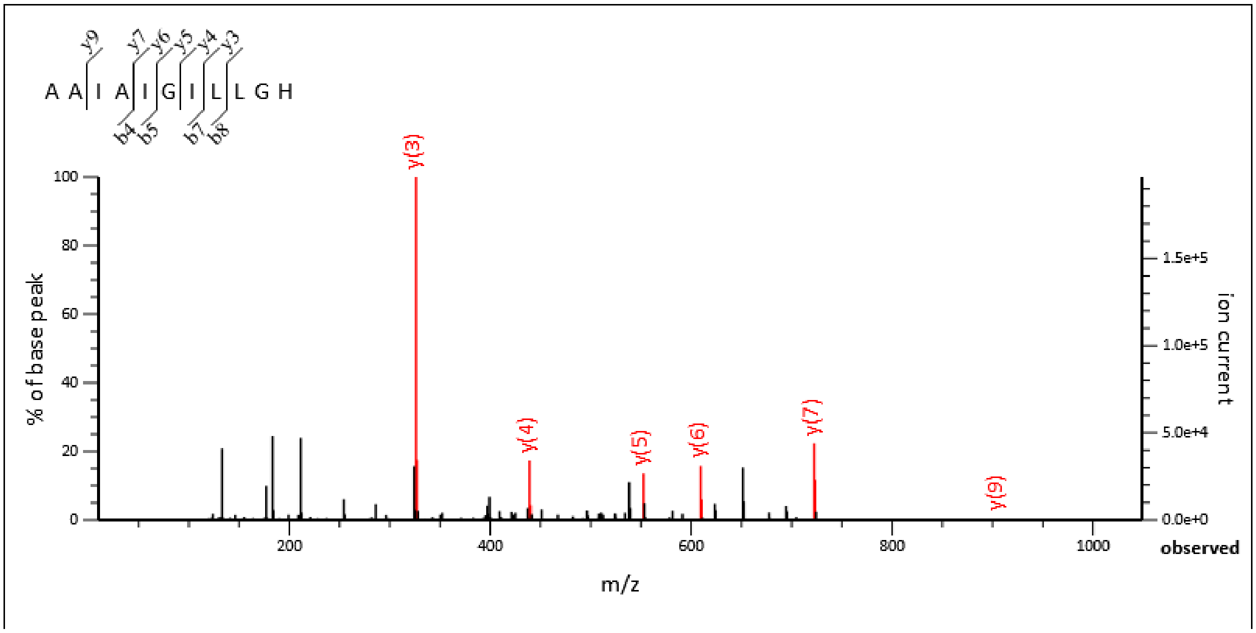

Peptidome-Bac 22

AITGLNDSPSY Mw (monoisotopic mass): 1136.53

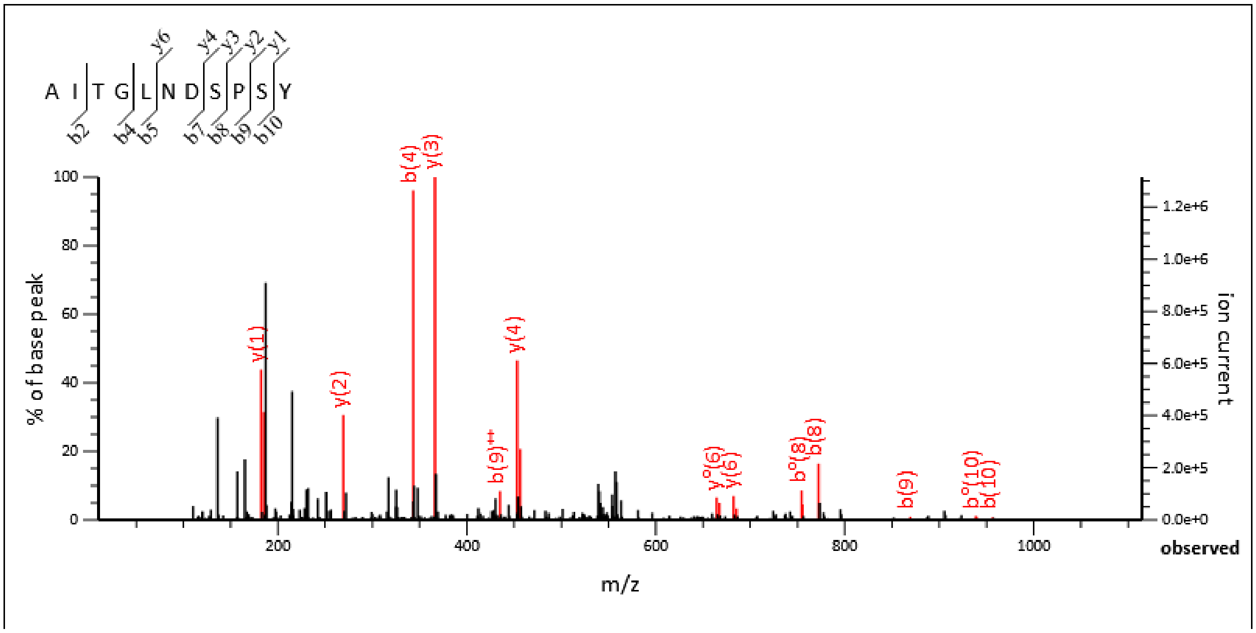

Peptidome-Bac 23

APEEILVVHDELDV Mw (monoisotopic mass): 2695.32

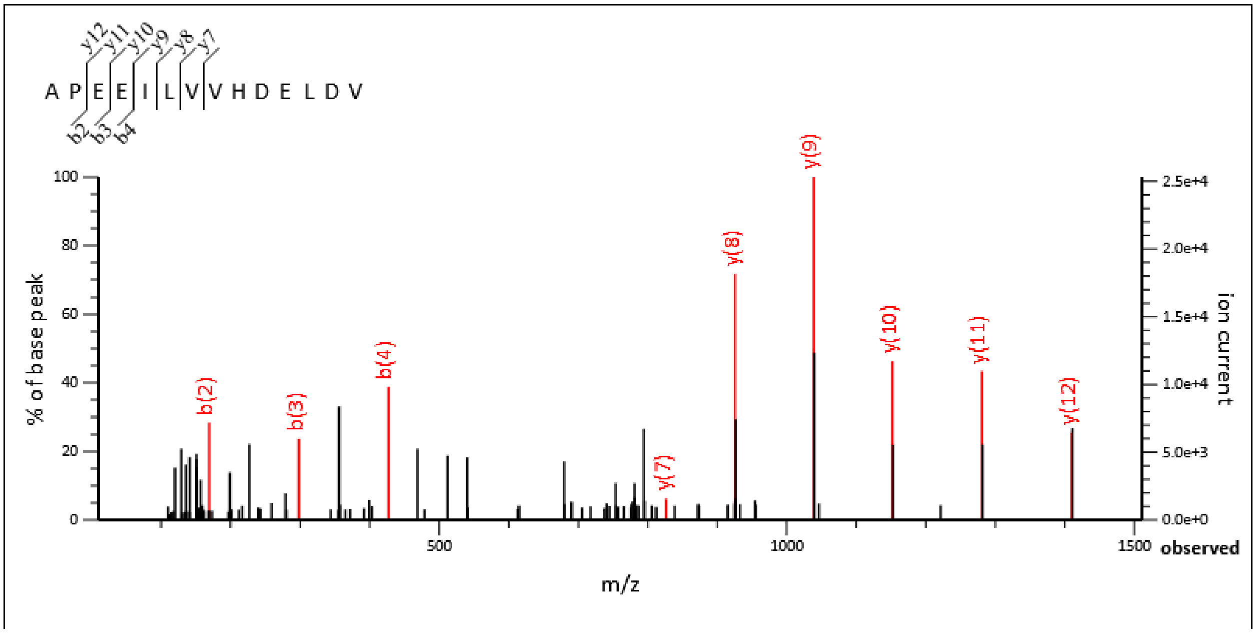

Peptidome-Bac 24

AVLDSLRFG Mw (monoisotopic mass): 976.53

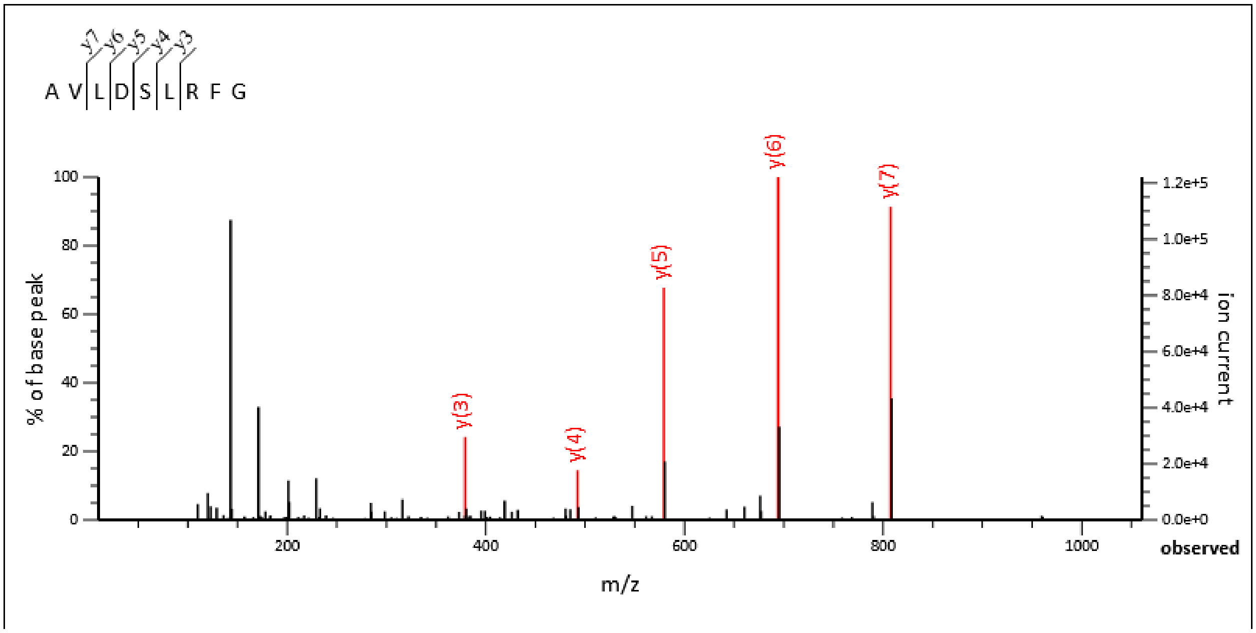

Peptidome-Bac 25

EDLLKEAF Mw (monoisotopic mass): 963.49

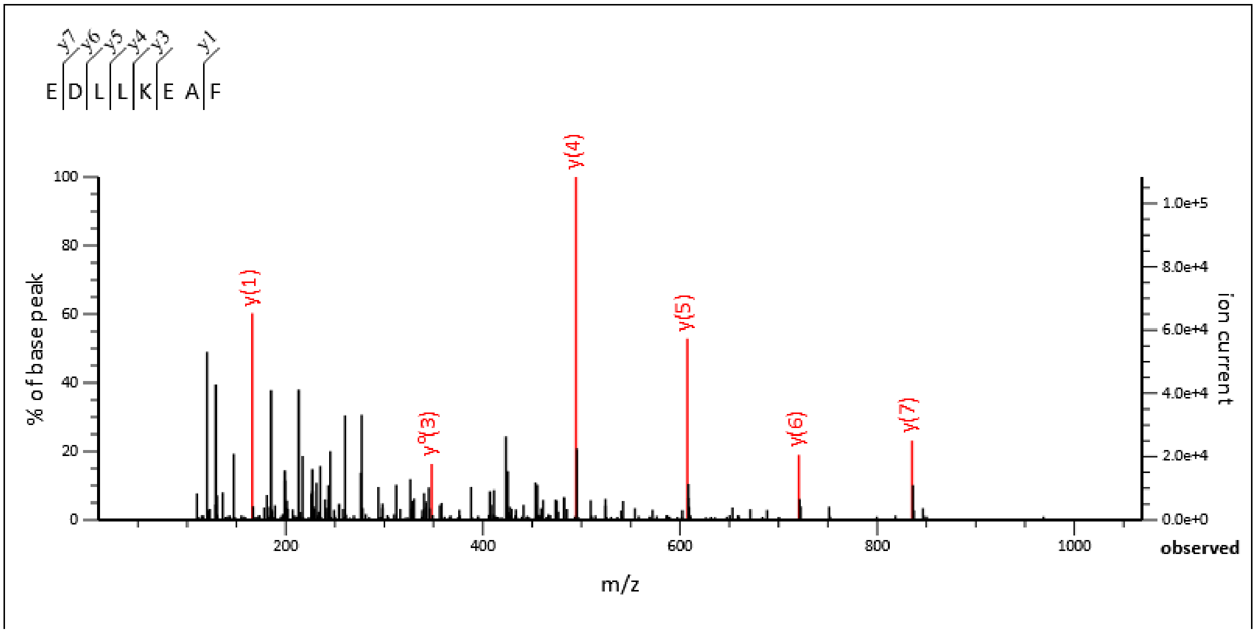

Peptidome-Bac 26

EGKVMLVDDVI Mw (monoisotopic mass): 1216.64

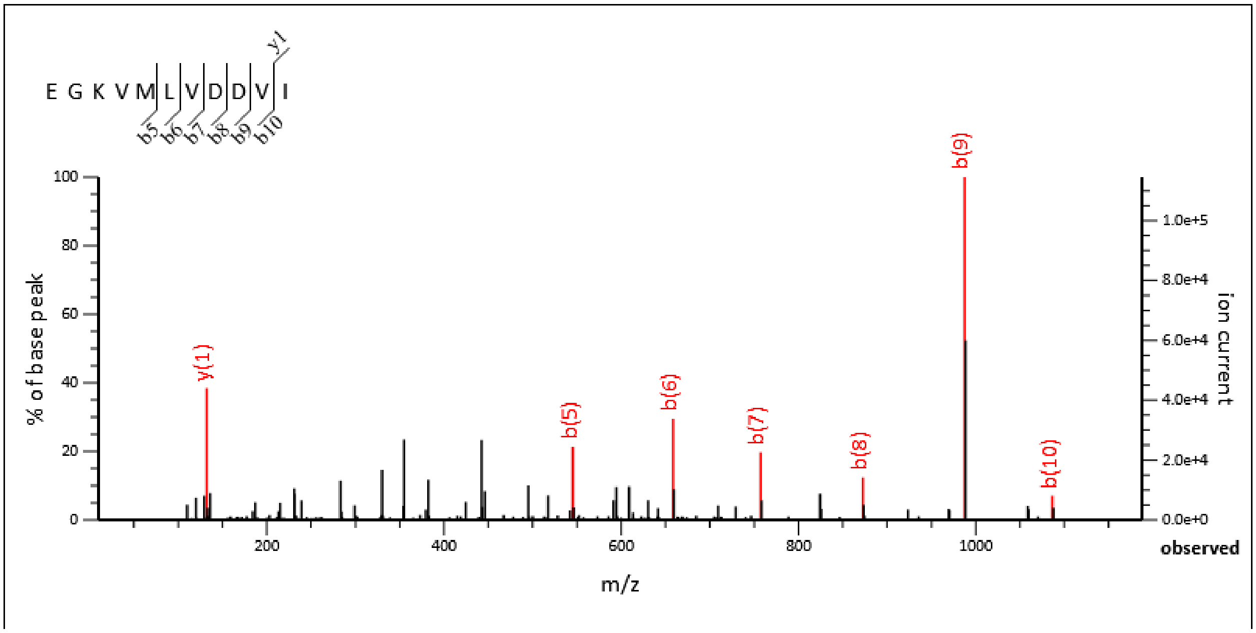

Peptidome-Bac 27

FVTLGGRT Mw (monoisotopic mass): 849.47

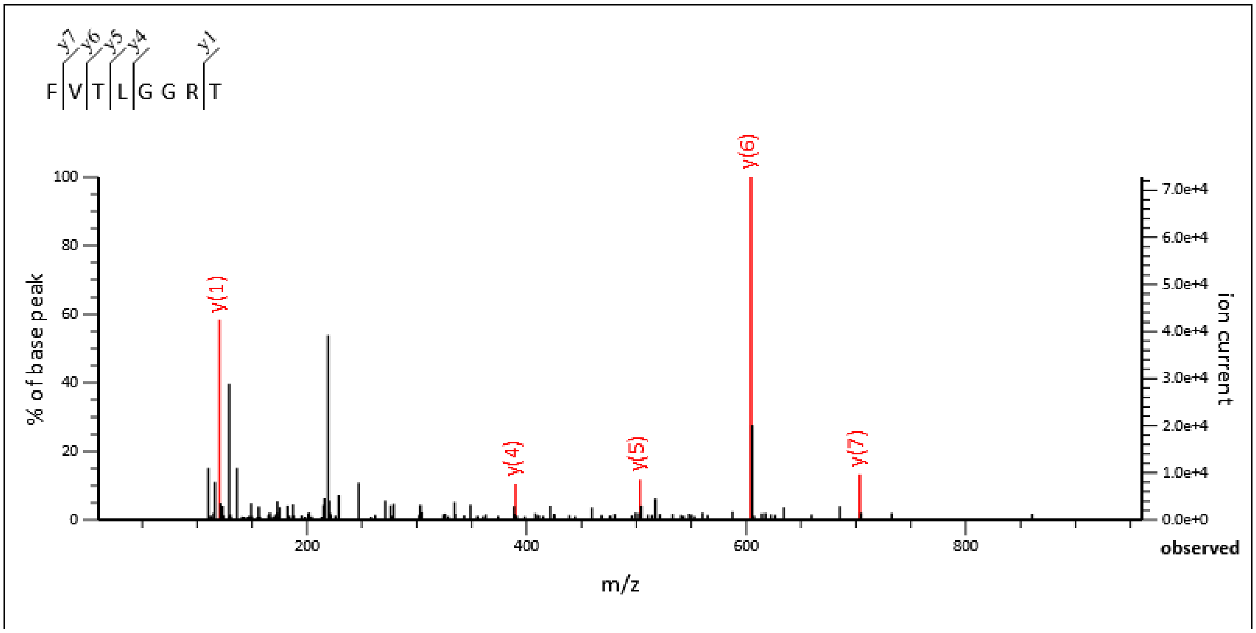

Peptidome-Bac 28

GAIGIGLLSL Mw (monoisotopic mass): 912.56

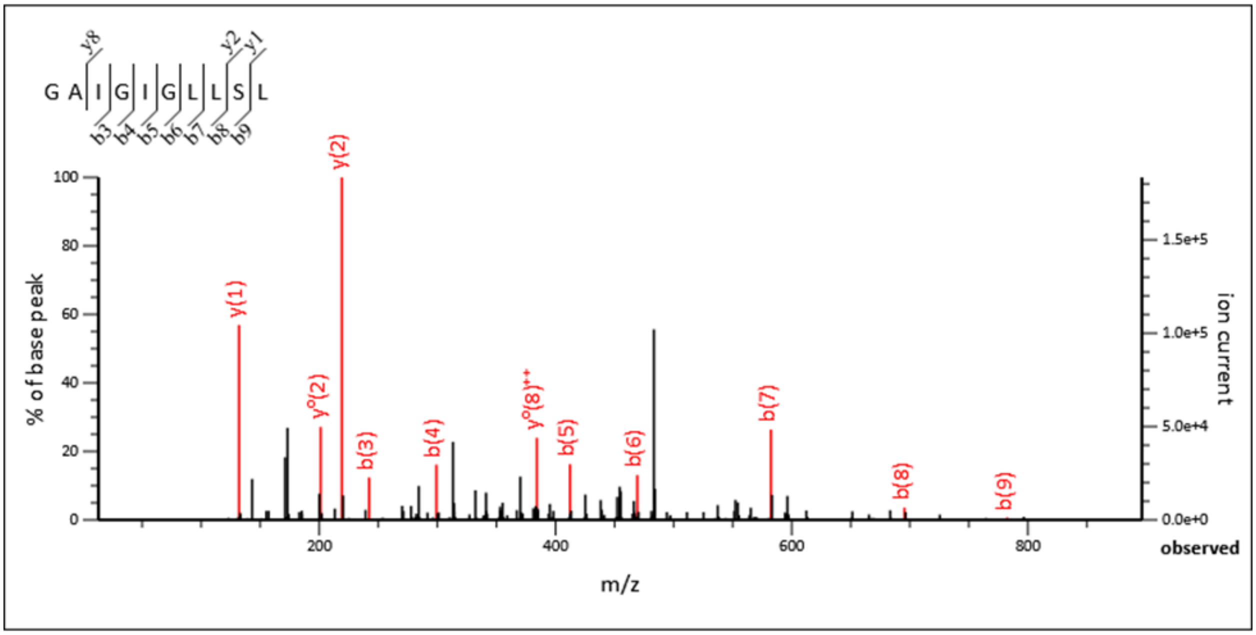

Peptidome-Bac 29

GQSAKLYIDDQYVGWLG Mw (monoisotopic mass): 1911.94

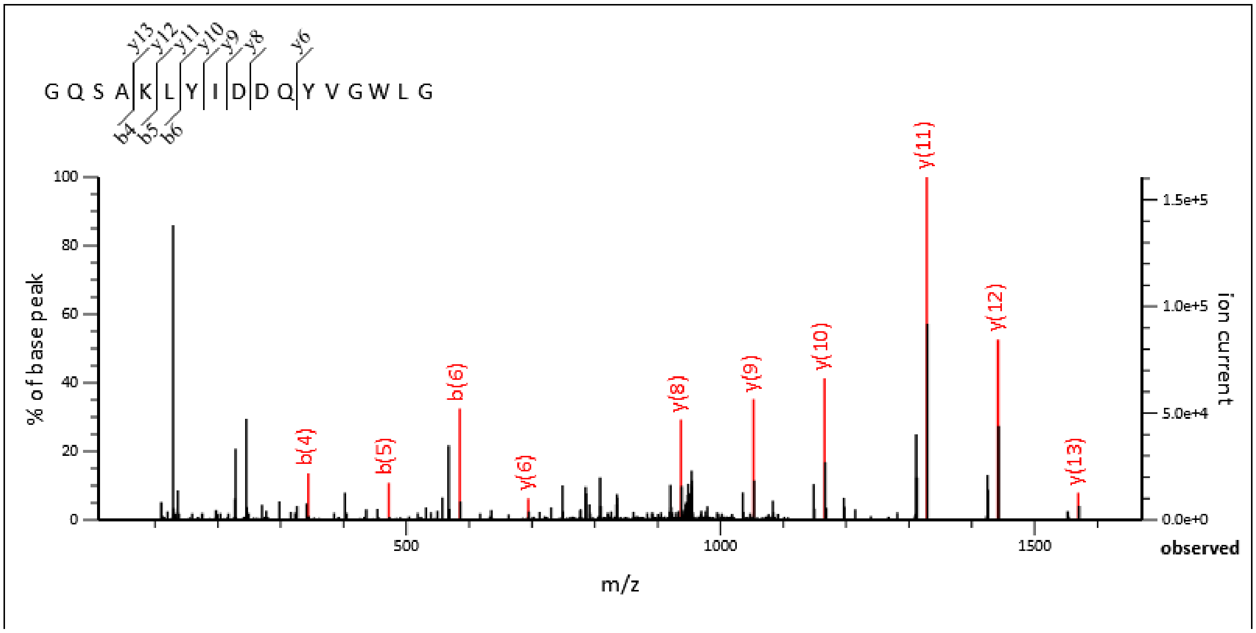

Peptidome-Bac 30

GVEDQLALFLPEDQPK Mw (monoisotopic mass): 1797.91

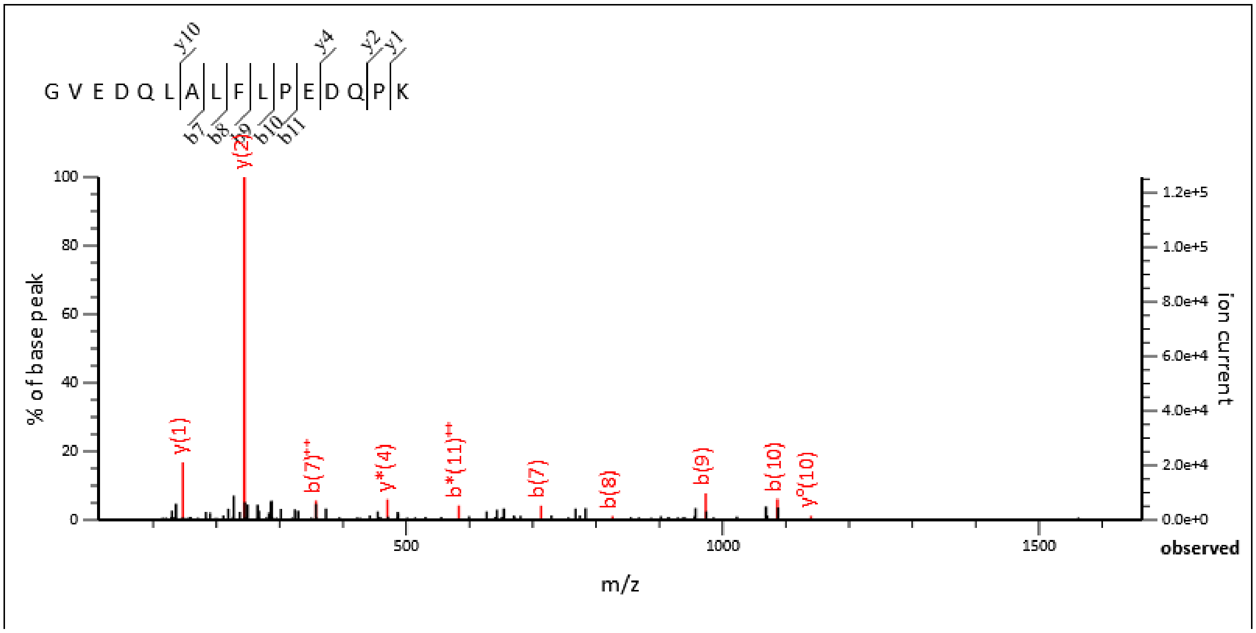

Peptidome-Bac 31

ISLTVDTDRLK Mw (monoisotopic mass): 1259.71

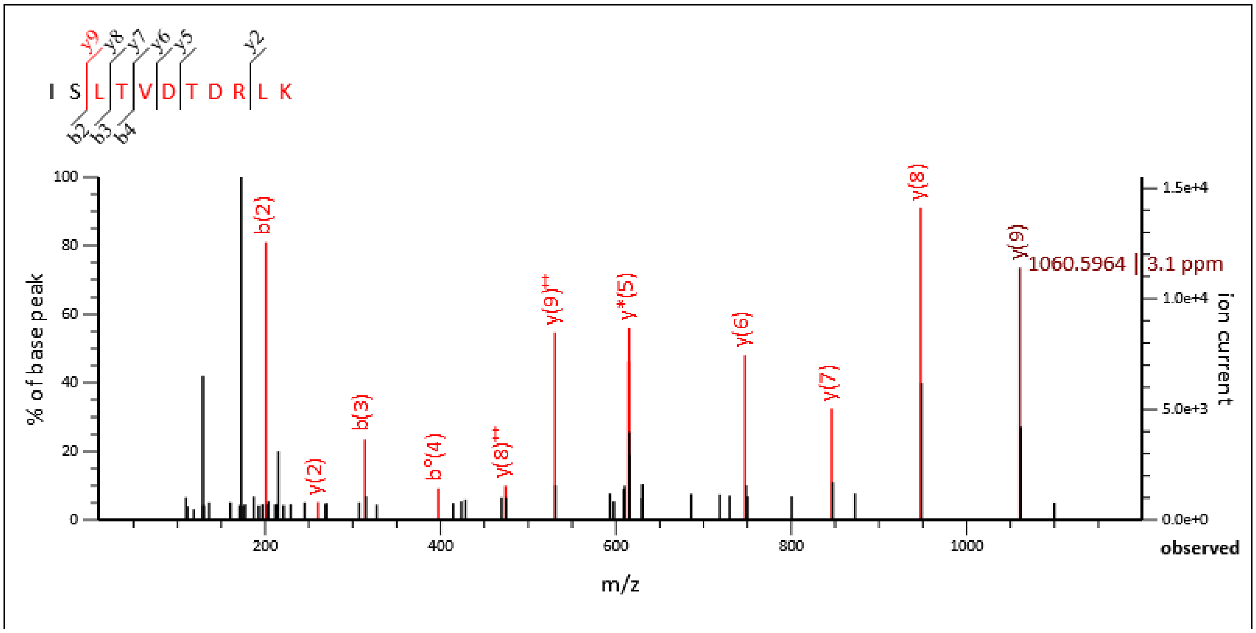

Peptidome-Bac 32

LSPPPSGVLSLCT Mw (monoisotopic mass): 1269.66

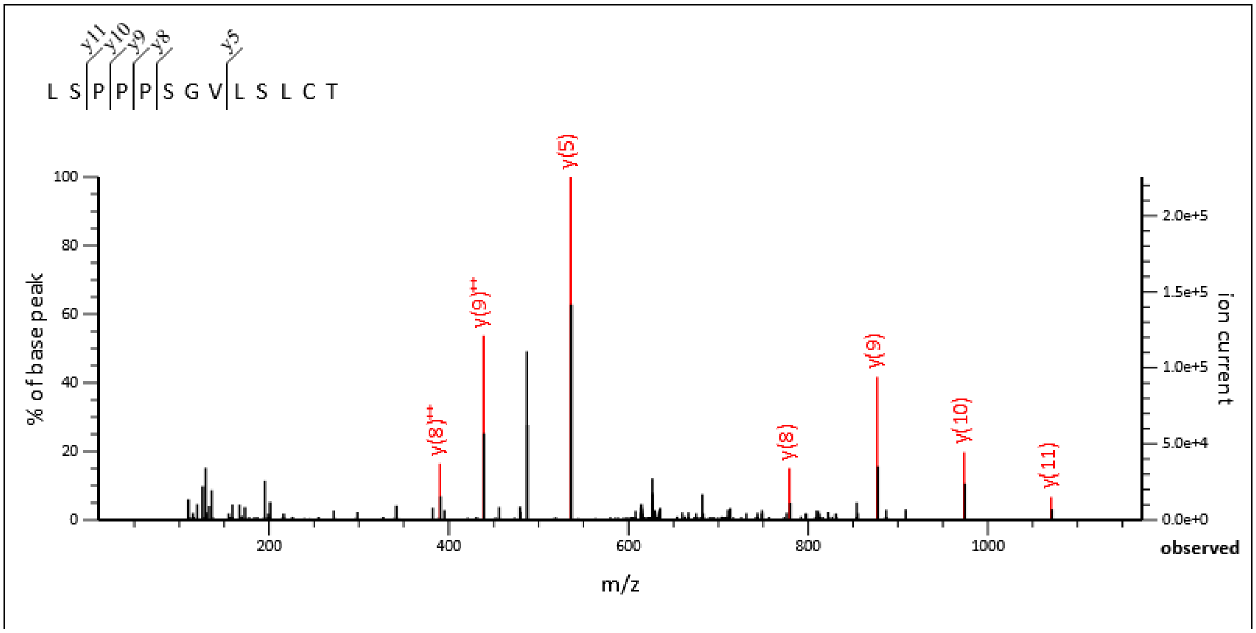

Peptidome-Bac 33

LYAPSLRGRRAIMLP Mw (monoisotopic mass): 1712.99

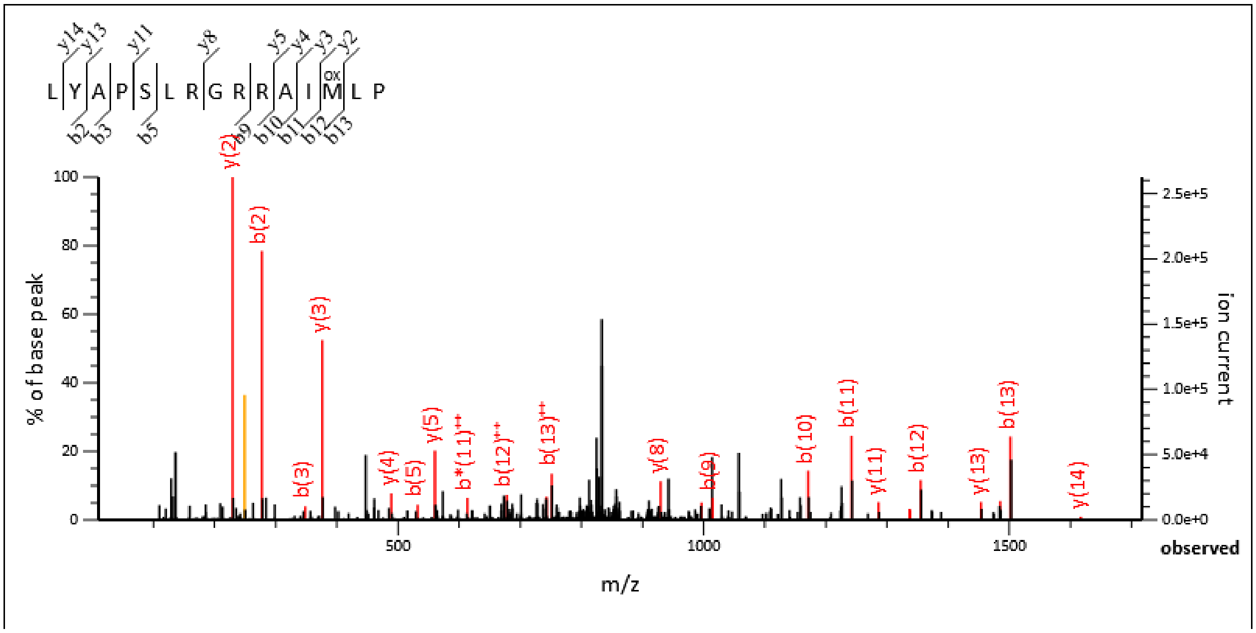

Peptidome-Bac 35

PVDILARSAAGETV Mw (monoisotopic mass): 1397.75

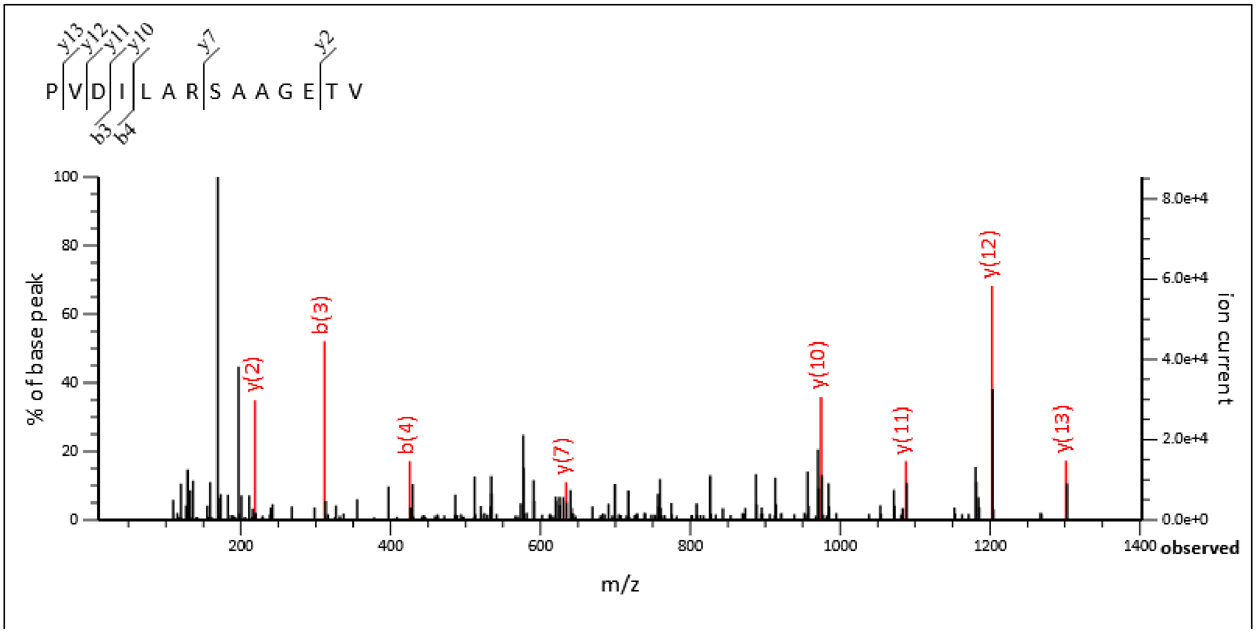

Peptidome-Bac 36

QIPFSSSSS Mw (monoisotopic mass): 938.43

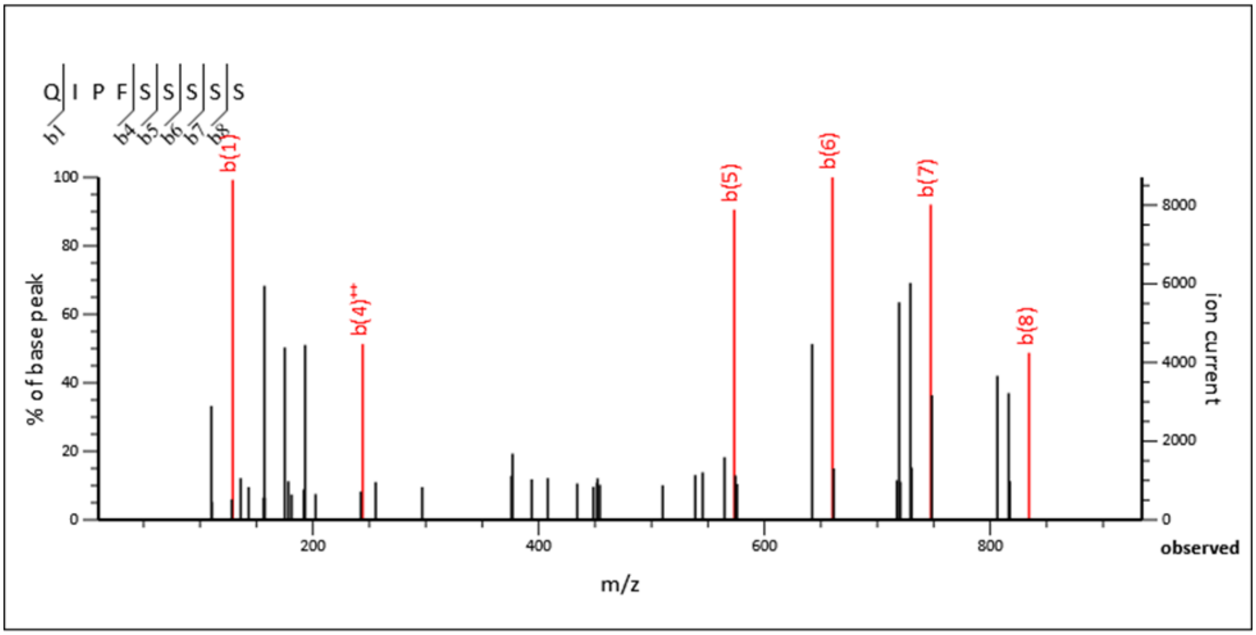

Peptidome-Bac 37

QLAGVLISL Mw (monoisotopic mass): 912.56

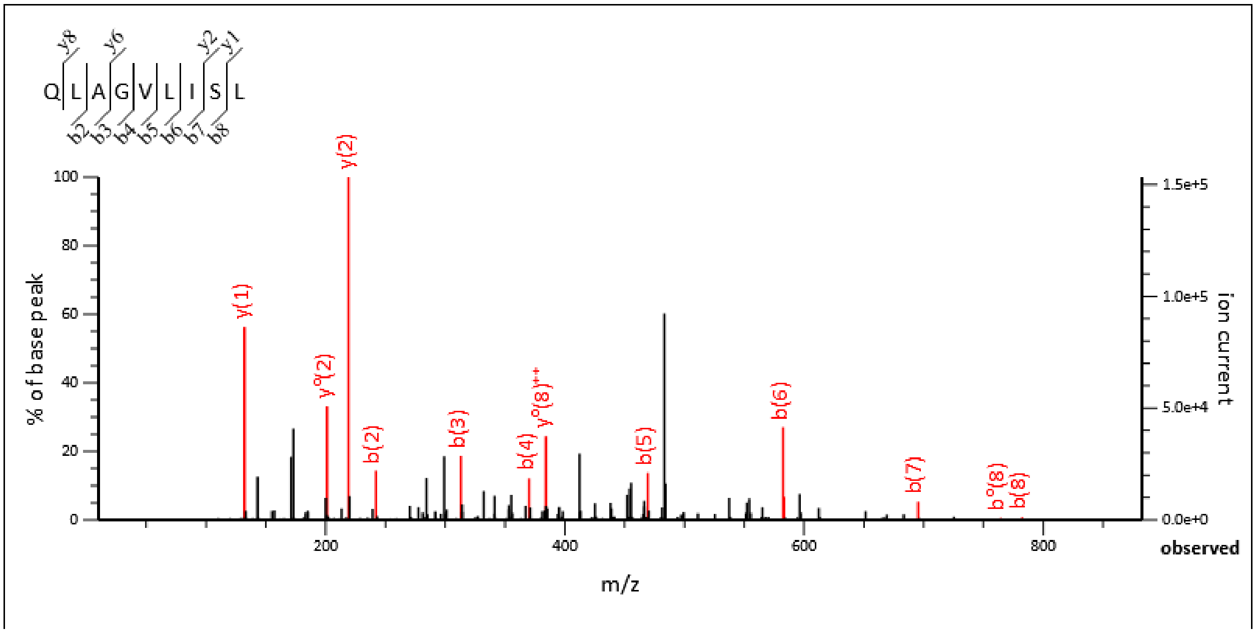

Peptidome-Bac 38

RDKVQDAITQKK Mw (monoisotopic mass): 1428.80

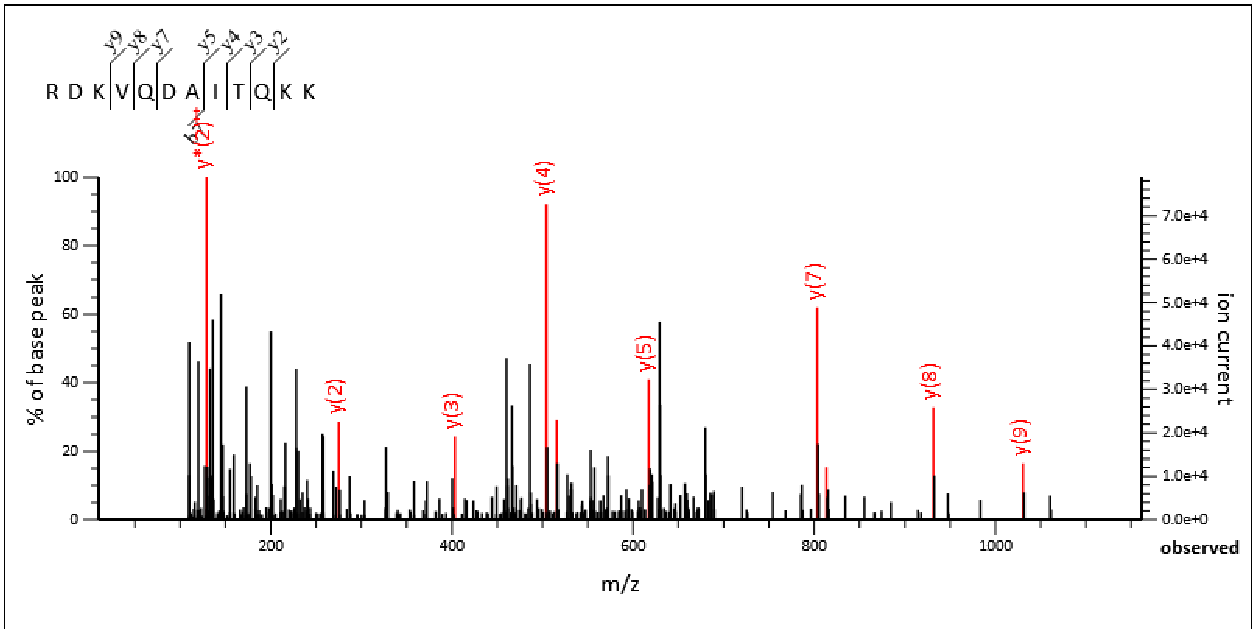

Peptidome-Bac 39

SDAQSNDNINKLQKL Mw (monoisotopic mass): 1686.85

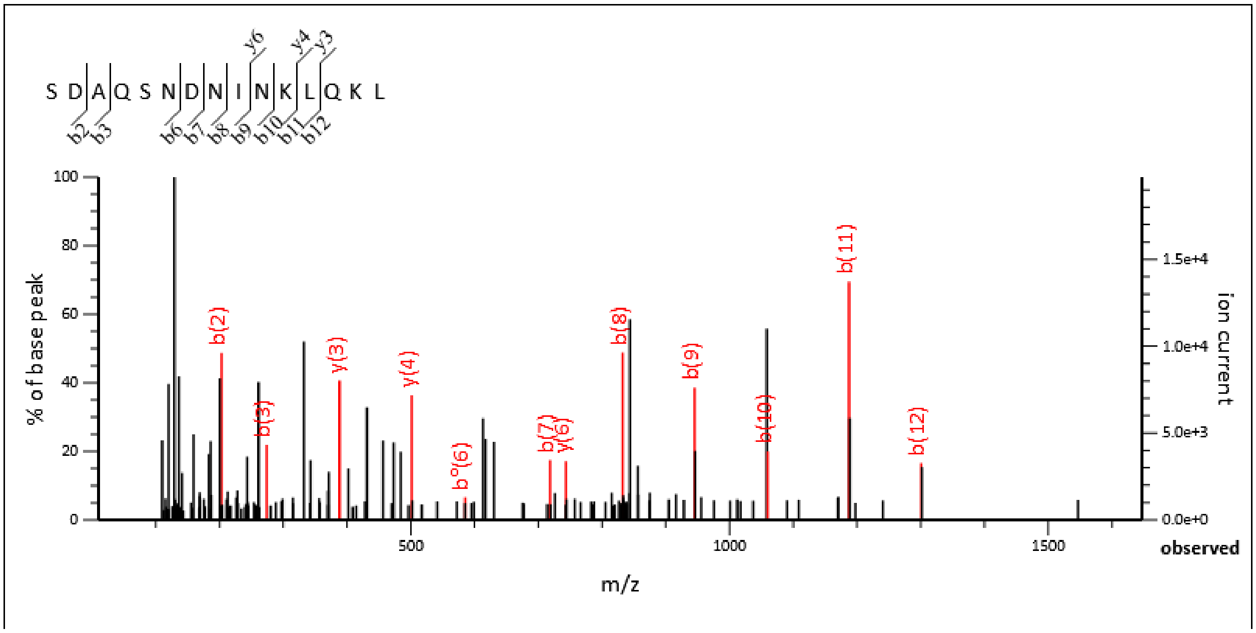

Peptidome-Bac 40

SEDEIVTEIVNLEAIL Mw (monoisotopic mass): 1785.92

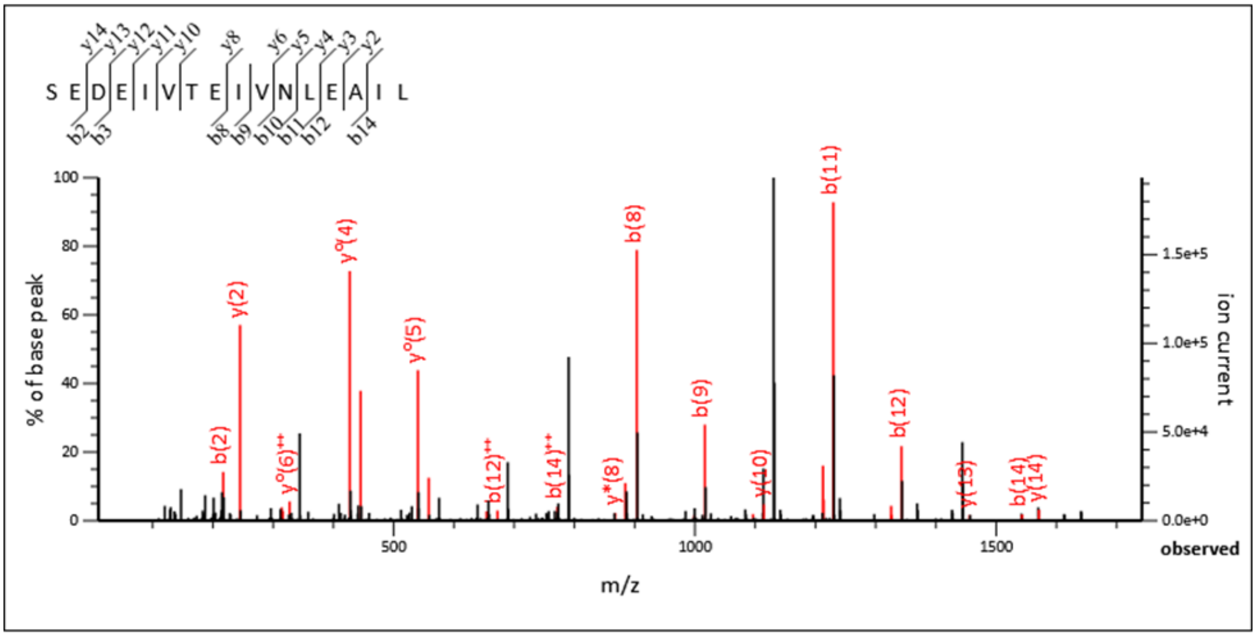

Supplementary Table 12. List of TCRBV antibodies and their corresponding IMGT annotation

TCRVB-PE		TCRVB-FITC	
Antibody	IMGT nomenclature	Antibody	IMGT nomenclature
TCR VB1	TRBV9	TCR VB3	TRBV28
TCR VB2	TRBV20-1	TCR VB5.1	TRBV5-1
TCR VB4	TRBV29-1	TCR VB5.2	TRBV5-6
TCR VB5.3	TRBV5-5	TCR VB8	TRBV12-3, TRBV12-4
TCR VB7.1	TRBV4-1, TRBV4-2, TRBV4-3	TCR VB11	TRBV25-1
TCR VB9	TRBV3-1	TCR VB13.6	TRBV6-6
TCR VB12	TRBV10-3	TCR VB16	TRBV14
TCR VB13.1	TRBV6-5, TRBV6-6, TRBV6-9	TCR VB17	TRBV19
TCR VB14	TRBV27	TCR VB21.3	TRBV11-2
TCR VB18	TRBV18	TCR VB22	TRBV2
TCR VB20	TRBV30		
TCR VB23	TRBV13		

Supplementary Table 13. Peptides used in vaccine and bacteria/microbiota pools

Vaccine pool	Bacteria/microbiota pool
**SEMA4D* peptide** (LQFVKDHPLMDDL)	**HB2 peptide** (QLEFNHAIAS)
**STC2* peptide** (RQLQVDRTKLSRA)	**HB4 peptide** (LWEFNNVSLA)
**SFRS17A peptide** (MGFIQAMSALRGM)	**HB84 peptide** (LIVFNHNCLK)
**PTPRA peptide** (DVFQTVKSLRLQR)	**HB101 peptide** (VWEFNHVPHK)
**LGI4 peptide** (GSFLRIPSLHLLL)	**HB124 peptide** (LLDFNFGPTR)
**LAMA5 peptide** (REFTKATNVRLRF)	**HB125 peptide** (IFEFNHSIGL)
**DNMT3A peptide** (LFFEFYRLLHDA)	**HGM2 peptide** (IWEFNHVPHK)
**PTCH2 peptide** (LEFAQFPFLLR)	**HGM3 peptide** (LLEFNHSLGM)
**LRP6 peptide** (HPFALTLFEDILY)	**HGM7 peptide** (LYEFNHSPGF)
**DOT1L peptide** (FKIQYLQFLAY)	**HGM14 peptide** (LYDFNHSSVK)
**CHD6 peptide** (KTFDWTQFRIISR)	**HGM21 peptide** (LYDFNHSAHV)
**SETBP1 peptide 1** (LQFLADLEELIT)	**HGM26 peptide** (LLDFNHSMVN)
**SETBP1 peptide 2** (LQFLADLEELITK)	**HGM27 peptide** (VWEFNHSLHP)
**CNTF peptide** (VAAFAYQIEELMI)	**HGM29 peptide** (TIDFNHGPTI)
**CHD7 peptide** (NKFLSEIEDELFN)	**HGM31 peptide** (LWDFAFSPSK)
**C5AR1* peptide** (WLVLGFLWPLLTL)	**HGM34 peptide** (VLEFNHGILK)
**GANC* peptide** (FRLKINEGTPLK)	**HGM45 peptide** (LYEFNHSTGK)
**SIN3A* peptide** (QPVEFNHAIHYVN)	**HGM56 peptide** (LMEFNHSPAF)
**ERBB2 peptide** (RLVHRDLAARNVL)	**HGM57 peptide** (MLEFNHVPLK)
**AIM2 peptide** (TLMIQNSGAVSAV)	**HGM62 peptide** (VFDFNHSAIK)
**PTPRZ1 peptide** (KHIRSQRNYLVQT)	**HGM66 peptide** (LLDFNFSPAS)
**NCOA5 peptide** (TGINFDNPSVQKA)	**HGM67 peptide** (LVEFNNSSLK)
	**HGM73 peptide** (LVEFNNSPMQ)

Supplementary Table 16. Comparing mass spectrometry spectra of 10 IPdBPs (top) with synthetic peptides (bottom) using Universal Spectrum Explorer

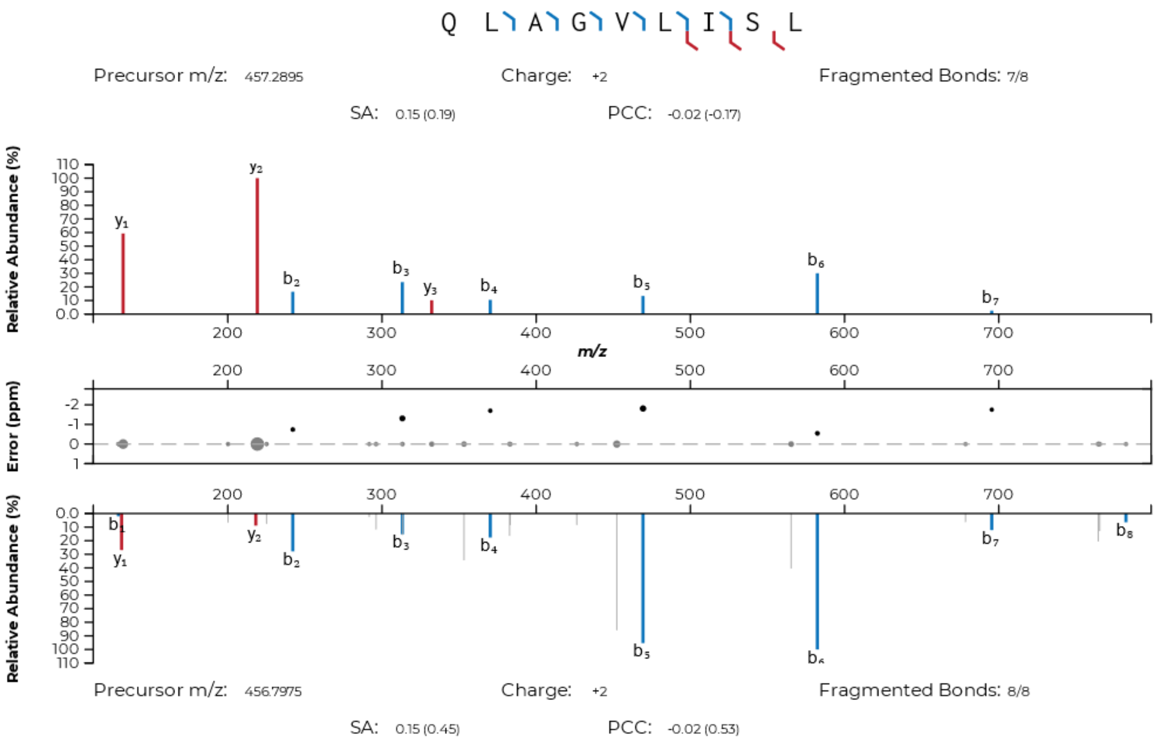

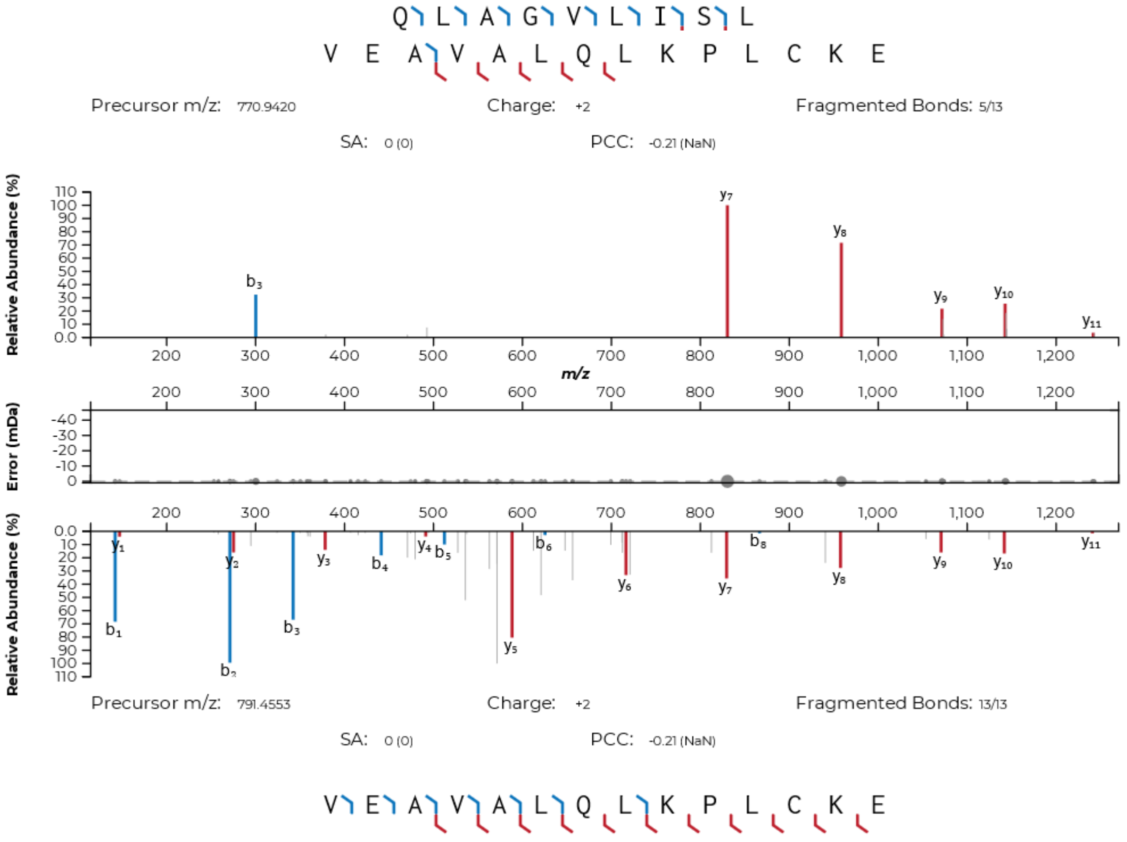

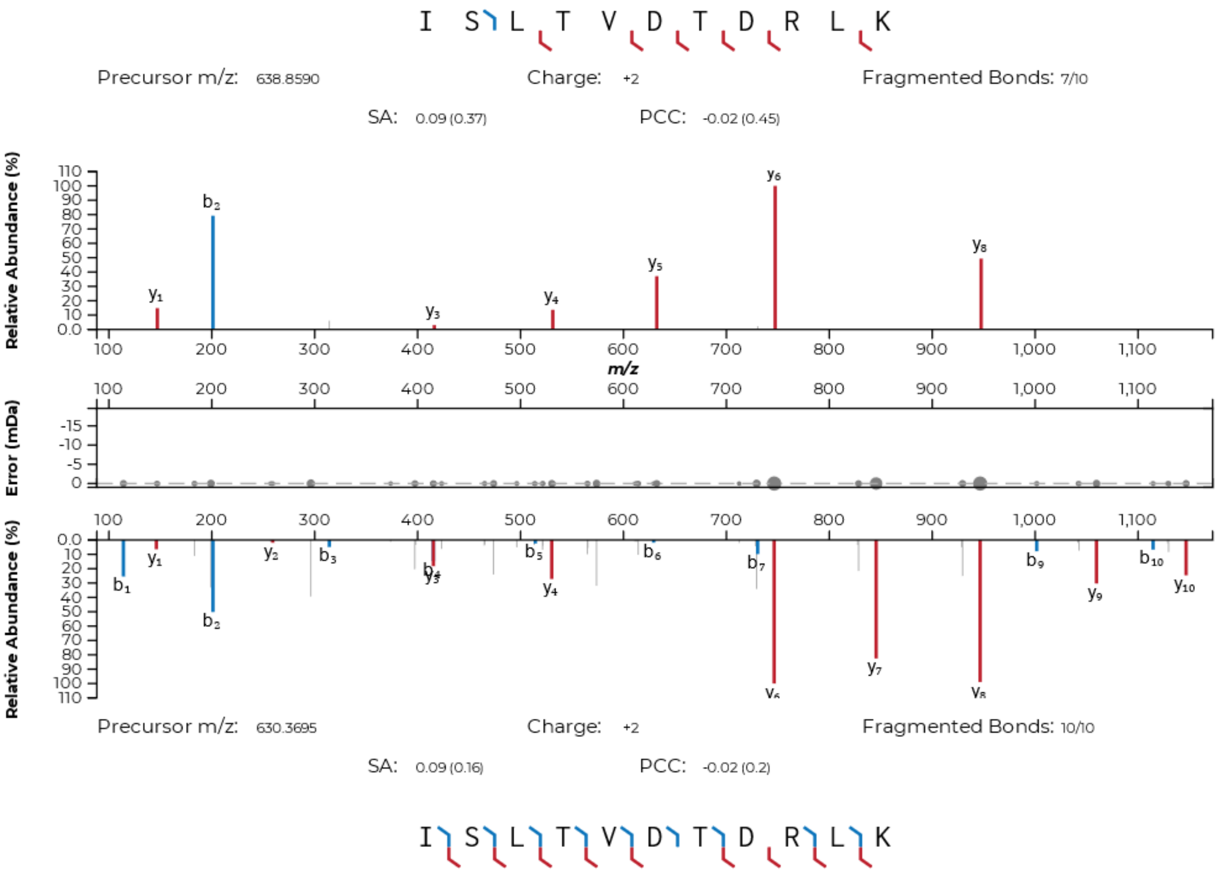
I S L T V D T D R L K
Precursor m/z: 638.8590
Charge: +2
Fragmented Bonds: 7/10
SA: 0.09 (0.37)
PCC: -0.02 (0.45)
Relative Abundance (%)
m/z
Error (mDa)
Relative Abundance (%)
Precursor m/z: 630.3695
Charge: +2
Fragmented Bonds: 10/10
SA: 0.09 (0.16)
PCC: -0.02 (0.2)
I S L T V D T D R L K

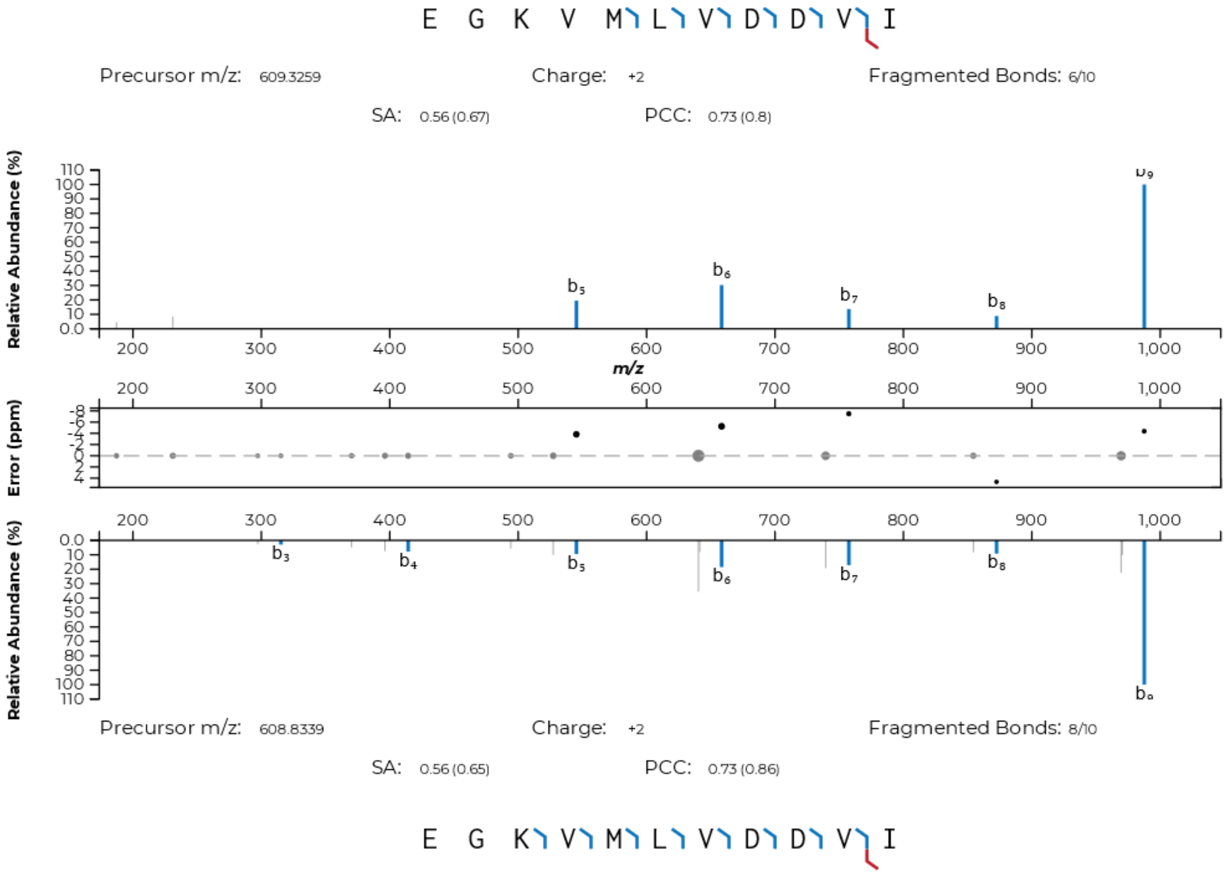
E G K V M L V D D V I
Precursor m/z: 609.3259
Charge: +2
Fragmented Bonds: 6/10
SA: 0.56 (0.67)
PCC: 0.73 (0.8)
Relative Abundance (%)
m/z
Error (ppm)
Relative Abundance (%)
Precursor m/z: 608.8339
Charge: +2
Fragmented Bonds: 8/10
SA: 0.56 (0.65)
PCC: 0.73 (0.86)
E G K V M L V D D V I

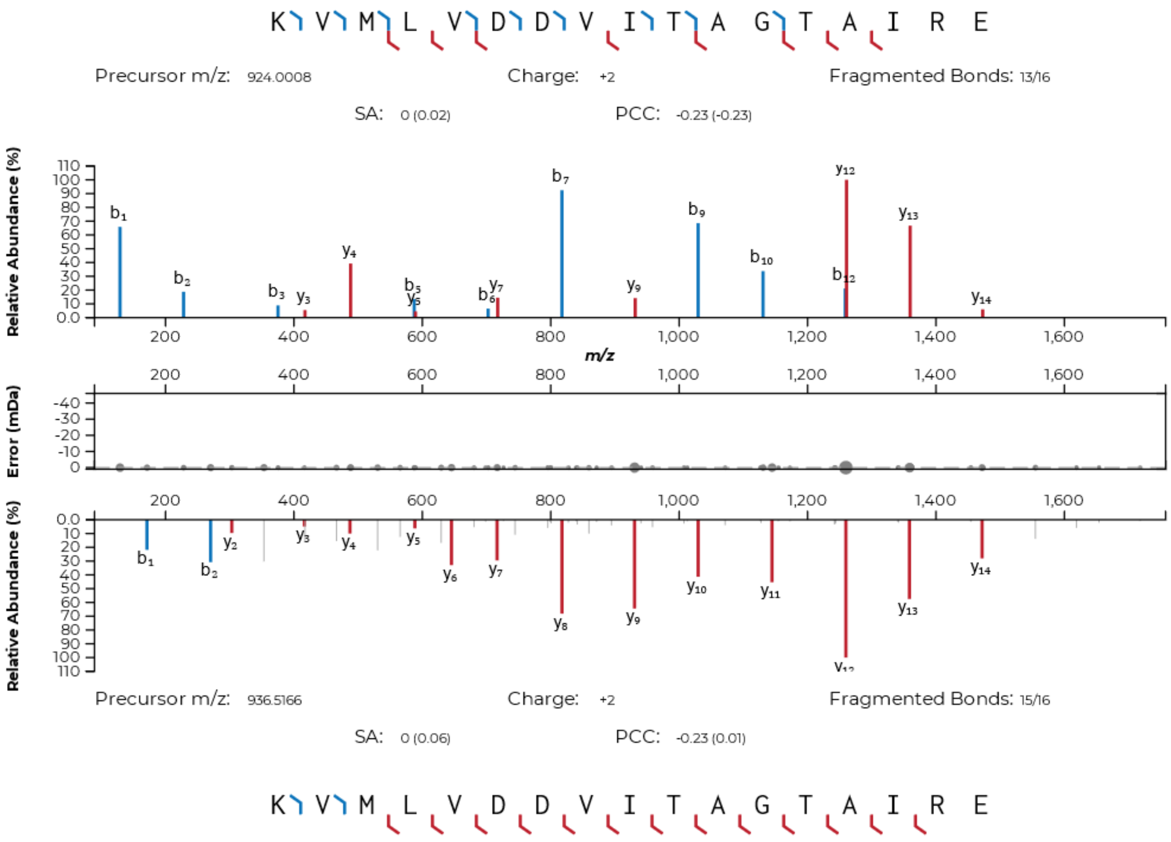
K V M L V D D V I T A G T A I R E
Precursor m/z: 924.0008
Charge: +2
Fragmented Bonds: 13/16
SA: 0 (0.02)
PCC: -0.23 (-0.23)
Relative Abundance (%)
m/z
Error (mDa)
Relative Abundance (%)
Precursor m/z: 936.5166
Charge: +2
Fragmented Bonds: 15/16
SA: 0 (0.06)
PCC: -0.23 (0.01)
K V M L V D D V I T A G T A I R E

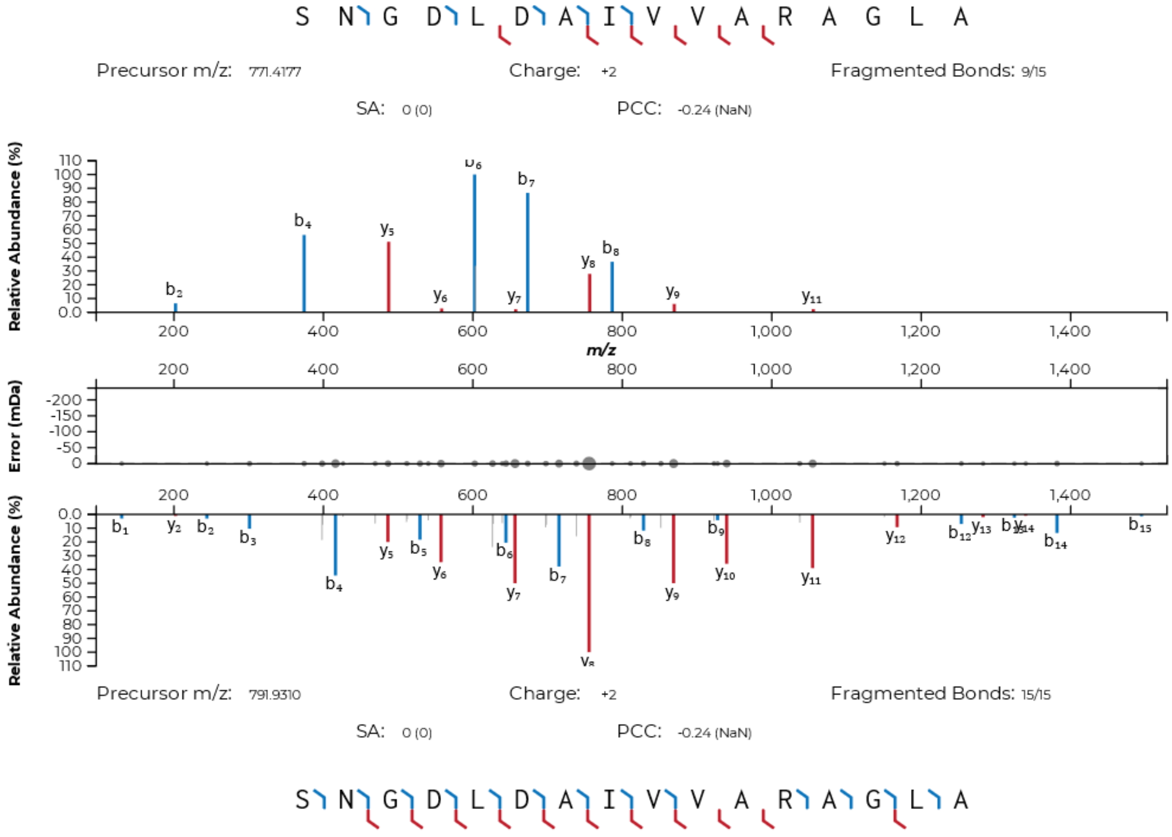
S N G D L D A I V V A R A G L A
Precursor m/z: 771.4177
Charge: +2
Fragmented Bonds: 9/15
SA: 0 (0)
PCC: -0.24 (NaN)
Relative Abundance (%)
m/z
Error (mDa)
Relative Abundance (%)
Precursor m/z: 791.9310
Charge: +2
Fragmented Bonds: 15/15
SA: 0 (0)
PCC: -0.24 (NaN)
S N G D L D A I V V A R A G L A

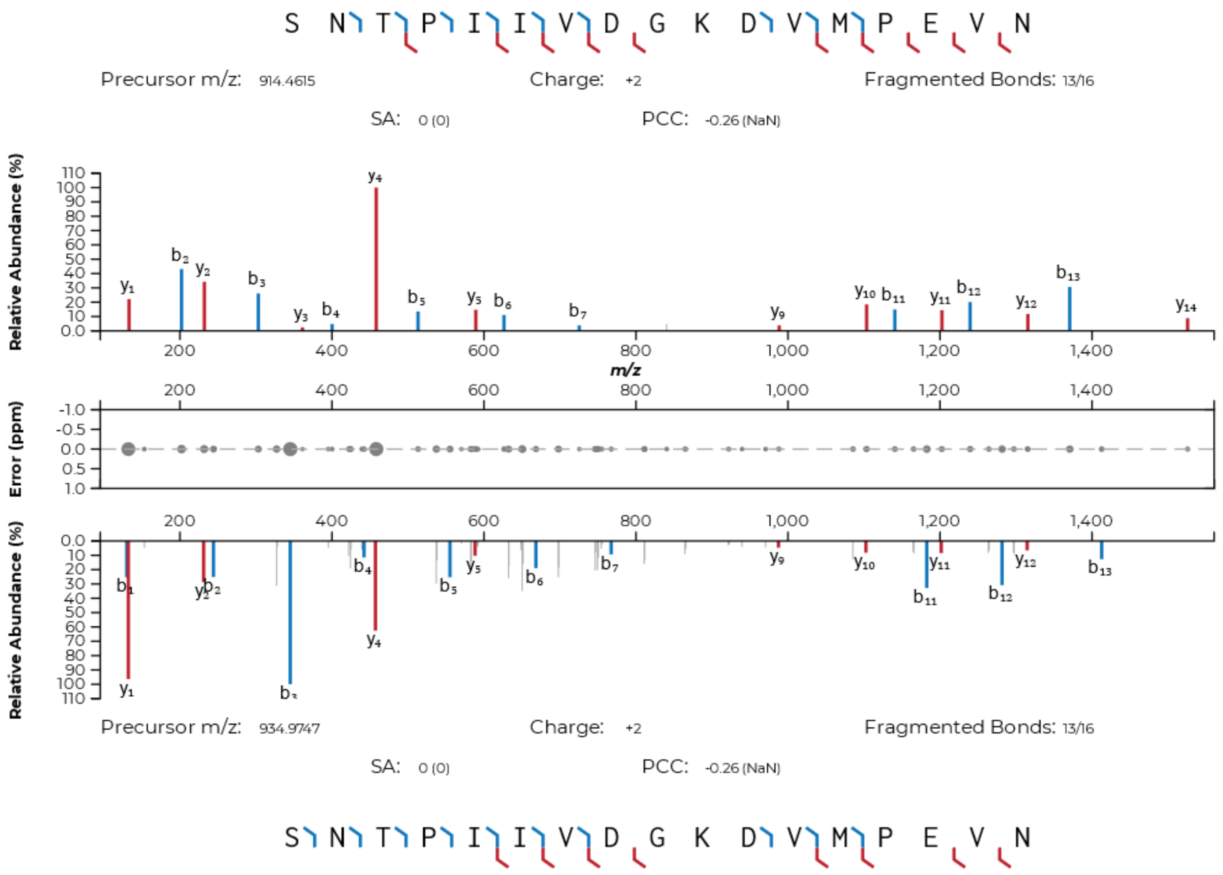
S N T P I I V D G K D V M P E V N
Precursor m/z: 914.4615
Charge: +2
Fragmented Bonds: 13/16
SA: 0 (0)
PCC: -0.26 (NaN)
Relative Abundance (%)
m/z
Error (ppm)
Relative Abundance (%)
Precursor m/z: 934.9747
Charge: +2
Fragmented Bonds: 13/16
SA: 0 (0)
PCC: -0.26 (NaN)
S N T P I I V D G K D V M P E V N

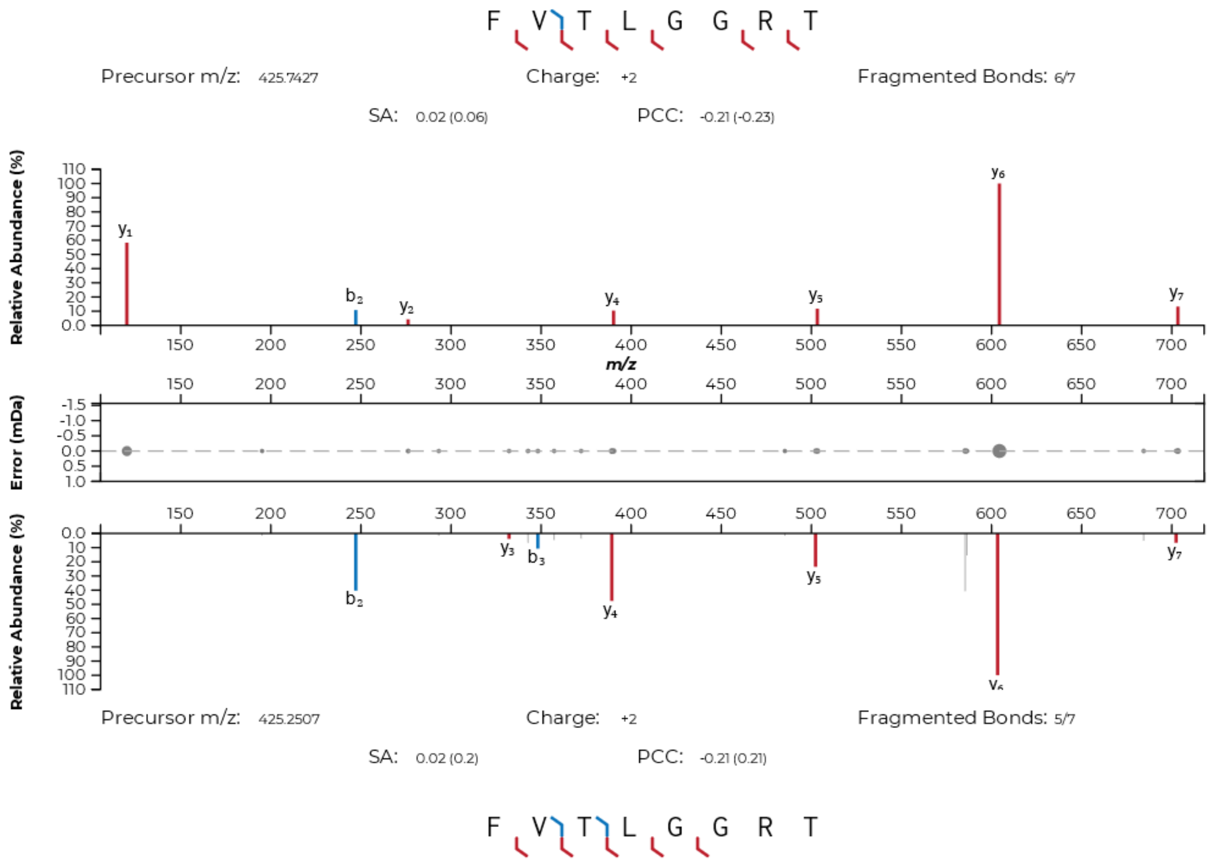
F V T L G G R T
Precursor m/z: 425.7427
Charge: +2
Fragmented Bonds: 6/7
SA: 0.02 (0.06)
PCC: -0.21 (-0.23)
Relative Abundance (%)
m/z
Error (mDa)
Relative Abundance (%)
Precursor m/z: 425.2507
Charge: +2
Fragmented Bonds: 5/7
SA: 0.02 (0.2)
PCC: -0.21 (0.21)
F V T L G G R T

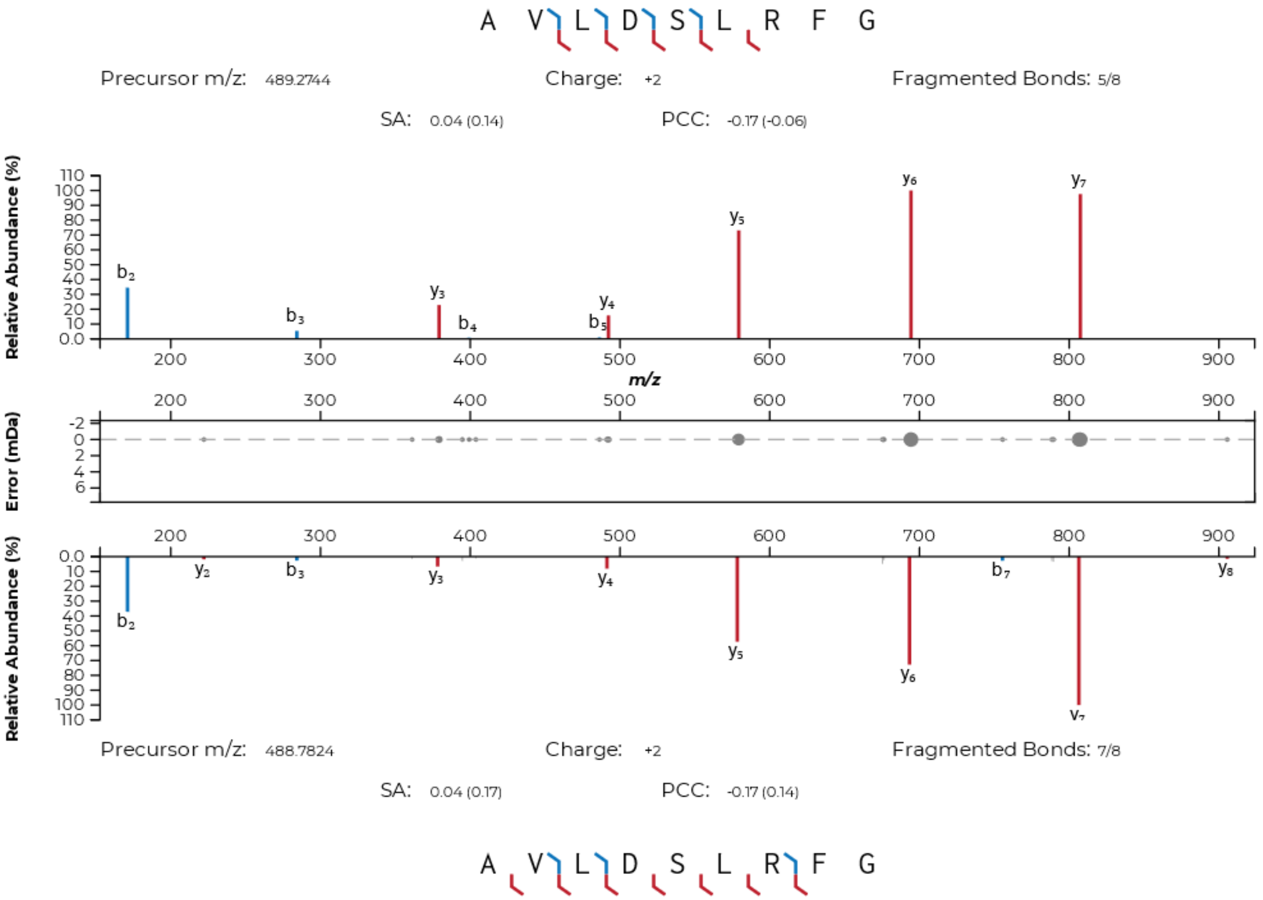
A V L D S L R F G
Precursor m/z: 489.2744
Charge: +2
Fragmented Bonds: 5/8
SA: 0.04 (0.14)
PCC: -0.17 (-0.06)
Relative Abundance (%)
m/z
Error (mDa)
Relative Abundance (%)
Precursor m/z: 488.7824
Charge: +2
Fragmented Bonds: 7/8
SA: 0.04 (0.17)
PCC: -0.17 (0.14)
A V L D S L R F G

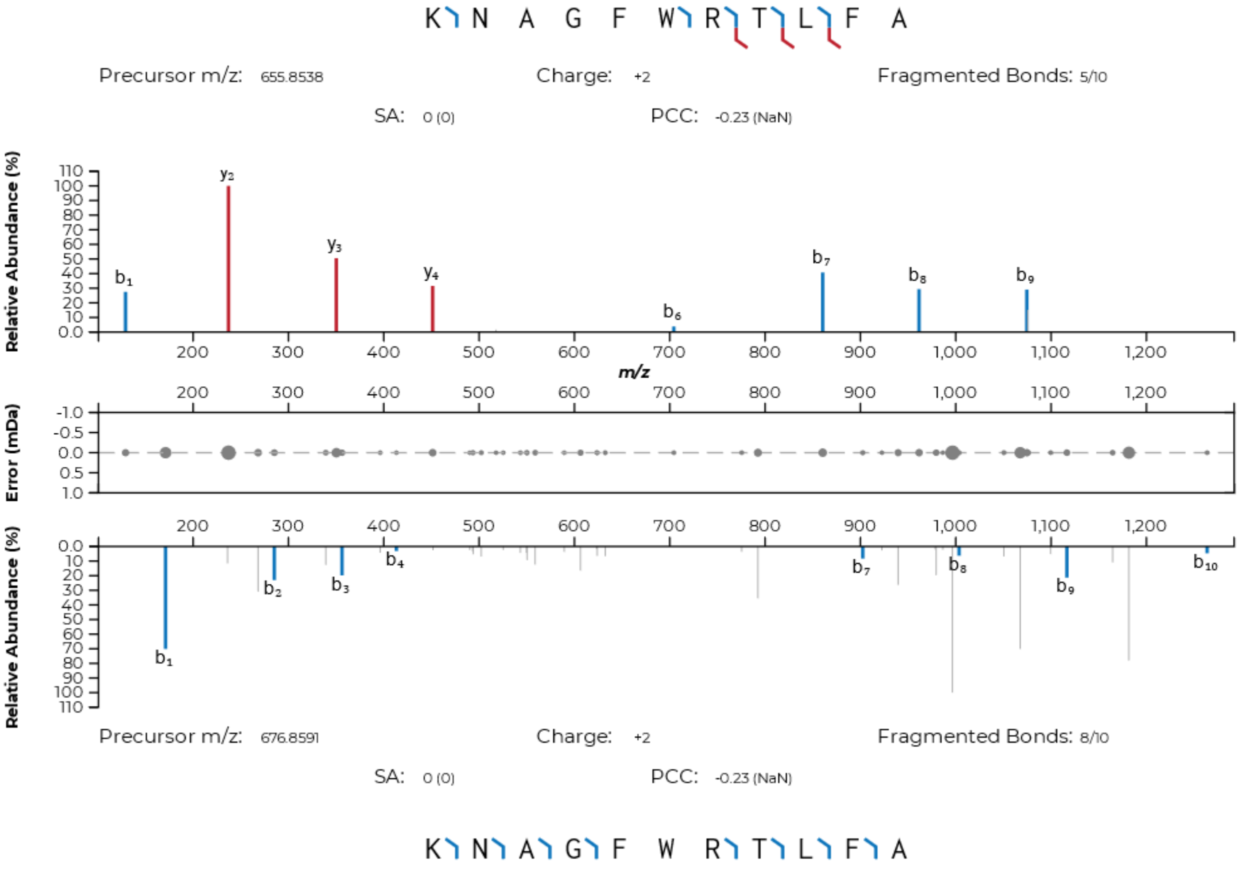
K N A G F W R T L F A
Precursor m/z: 655.8538
Charge: +2
Fragmented Bonds: 5/10
SA: 0 (0)
PCC: -0.23 (NaN)
Relative Abundance (%)
m/z
Error (mDa)
Relative Abundance (%)
Precursor m/z: 676.8591
Charge: +2
Fragmented Bonds: 8/10
SA: 0 (0)
PCC: -0.23 (NaN)
K N A G F W R T L F A
