# Supplementary material for: Microbial peptides activate tumour-infiltrating lymphocytes in glioblastoma
Source: Nature. 2023 May 17;617(7962):807–17. doi: 10.1038/s41586-023-06081-w (PMC10208956; doi:10.1038/s41586-023-06081-w)
Supplement: Supplementary file 2 — Reporting Summary [file 41586_2023_6081_MOESM2_ESM.pdf]

nature portfolio

Corresponding author(s): Roland Martin

Last updated by author(s): Feb 21, 2023

Reporting Summary

Nature Portfolio wishes to improve the reproducibility of the work that we publish. This form provides structure for consistency and transparency in reporting. For further information on Nature Portfolio policies, see our Editorial Policies and the Editorial Policy Checklist.

Statistics

For all statistical analyses, confirm that the following items are present in the figure legend, table legend, main text, or Methods section.

n/a	Confirmed	
☐	☒	The exact sample size (*n*) for each experimental group/condition, given as a discrete number and unit of measurement
☐	☒	A statement on whether measurements were taken from distinct samples or whether the same sample was measured repeatedly
☐	☒	The statistical test(s) used AND whether they are one- or two-sided<br>*Only common tests should be described solely by name; describe more complex techniques in the Methods section.*
☐	☒	A description of all covariates tested
☐	☒	A description of any assumptions or corrections, such as tests of normality and adjustment for multiple comparisons
☐	☒	A full description of the statistical parameters including central tendency (e.g. means) or other basic estimates (e.g. regression coefficient) AND variation (e.g. standard deviation) or associated estimates of uncertainty (e.g. confidence intervals)
☐	☒	For null hypothesis testing, the test statistic (e.g. *F*, *t*, *r*) with confidence intervals, effect sizes, degrees of freedom and *P* value noted<br>*Give P values as exact values whenever suitable.*
☒	☐	For Bayesian analysis, information on the choice of priors and Markov chain Monte Carlo settings
☒	☐	For hierarchical and complex designs, identification of the appropriate level for tests and full reporting of outcomes
☒	☐	Estimates of effect sizes (e.g. Cohen's *d*, Pearson's *r*), indicating how they were calculated

Our web collection on statistics for biologists contains articles on many of the points above.

Software and code

Policy information about availability of computer code

Data collection	No software was used for data collection
Data analysis	GraphPad Prism 8, IceLogo (https://iomics.ugent.be/icelogoserver/) , FlowJo_v10.8.1, VENNY2.1, Scaffold version 5.2, Proteome Software, MASCOT software (https://proteomicsresource.washington.edu/mascot/cgi/login.pl), Universal Spectrum Explorer (https://www.proteomicsdb.org/use/), Proteome Discoverer (Thermo Fischer), bcl2fastq v2.20.0.422, FastQC v 0.11.8, cutadapt v3.2, USEARCH v. 11.0.667, NetMHCII 2.3, Biorender (https://biorender.com/)

For manuscripts utilizing custom algorithms or software that are central to the research but not yet described in published literature, software must be made available to editors and reviewers. We strongly encourage code deposition in a community repository (e.g. GitHub). See the Nature Portfolio guidelines for submitting code & software for further information.

Data

Policy information about availability of data

All manuscripts must include a data availability statement. This statement should provide the following information, where applicable:

- Accession codes, unique identifiers, or web links for publicly available datasets
- A description of any restrictions on data availability
- For clinical datasets or third party data, please ensure that the statement adheres to our policy

The TCRVB sequencing results reported in this paper are publicly available via the immuneACCESS database of Adaptive Biotechnologies: (https://

clients.adaptivebiotech.com/pub/naghavian-2023-n)
The mass spectrometry immunopeptidomic raw data have been deposited to the ProteomeXchange Consortium via the PRIDE partner repository with the dataset identifier PXD036811 (https://www.ebi.ac.uk/pride/archive/projects/PXD036811)
The Cancer Genome Atlas (TCGA) GBM cohort downloaded from the Broad GDAC Firehose (Broad Institute of MIT and Harvard. doi:10.7908/C16T0M0N). Uniprot human proteins (Swiss-Prot-20201202) (https://www.uniprot.org/proteomes?query=(taxonomy_id:9606) , Uniprot viruses (Swiss-Prot-20210305) https://www.uniprot.org/uniprotkb?query=viruses%20AND%20(virus_host_id:9606), Uniprot bacteria (Swiss-Prot-20201202) (https://www.uniprot.org/proteomes/?facets=superkingdom%3ABacteria&query=%2A) and human gut microbiota protein database (Wen, P. Y. et al. 2020)

Human research participants

Policy information about studies involving human research participants and Sex and Gender in Research.

Reporting on sex and gender	Sex and gender were collected upon self-reports. Since our study deeply focuses on understanding the immunogenicity of commensal and pathogenic bacteria antigens on many T cell clones isolated from a patient underwent a neoantigen vaccination therapy, sex and gender was not considered in the study design.
Population characteristics	In total, 20 patients (13 males, 7 females) were included. The mean age was 66.75 years (range 46-86). All patients were IDH1 wildtype. These information are available in supplementary tables 1, 2.
Recruitment	All included patients underwent elective brain tumor surgery at the Department of Neurosurgery at the University Hospital Zurich and were diagnosed with glioblastoma (IDH1 wildtype) by a board-certified neuropathologist. No other inclusion or exclusion criteria were applied so that we do not expect other biases of any kind. Freshly resected human tissue and blood samples were obtained from the Department of Neurosurgery at the University Hospital Zurich. Written informed consent was obtained from each patient in accordance with the local ethical requirements (KEK-ZH-Nr. 2015-0163). Patient 1635WI was interested in participating in an individual treatment attempt with a personalized peptide vaccination therapy (Wang et al., submitted 2022). He was fully informed about the potential risks of such a vaccination, which by definition is an individual medical treatment (“compassionate use“) and not subject to special regulations for medical research according to the Therapeutic Products Act (TPA) or Swiss Federal Human Research Act (HRA). Consequently, individual medical treatments are not approved by the Ethics Committee or Swiss Agency for Therapeutic Product (Swissmedic), as they do not constitute clinical trials for the purpose of systematically gaining knowledge. The patient gave written informed consent to receive the individualized vaccination.
Ethics oversight	Written informed consent was obtained from each patient in accordance with the local ethical requirements, Zurich, Switzerland (KEK-ZH-Nr. 2015-0163).

Note that full information on the approval of the study protocol must also be provided in the manuscript.

Field-specific reporting

Please select the one below that is the best fit for your research. If you are not sure, read the appropriate sections before making your selection.

☒ Life sciences ☐ Behavioural & social sciences ☐ Ecological, evolutionary & environmental sciences

For a reference copy of the document with all sections, see nature.com/documents/nr-reporting-summary-flat.pdf

Life sciences study design

All studies must disclose on these points even when the disclosure is negative.

Sample size	Sample size was determined based on the availability of biological materials
Data exclusions	No data was excluded.
Replication	All experiments including immunopeptidome, bulk sorted T cells in response to vaccine, bacteria/microbiota-derived peptide pools, TCC88's response to ps_SCL were conducted in at least 3 replicates and were all also reproducible. Cytotoxicity assay, HLA-DR expression of tumor cells and staining of the tumor tissue were done in duplicates as we had limited access to certain material. TCCs large screening with bacteria/ microbiota-derived peptides were also conducted as duplicates. All experiments were reproducible.
Randomization	Randomization is not relevant for this study. The study was not a controlled clinical trial, in which an intervention had been compared to a control (either placebo or verum), but rather used biomaterials (i.e. tumor tissue) from glioblastoma patients, who had received surgery.
Blinding	Blinidng is not relevant for this study. The study was not a controlled clinical trial, in which an intervention had been compared to a control (either placebo or verum), but rather used biomaterials (i.e. tumor tissue) from glioblastoma patients, who had received surgery.

Reporting for specific materials, systems and methods

We require information from authors about some types of materials, experimental systems and methods used in many studies. Here, indicate whether each material, system or method listed is relevant to your study. If you are not sure if a list item applies to your research, read the appropriate section before selecting a response.

Materials & experimental systems

n/a	Involved in the study
☐	☒ Antibodies
☐	☒ Eukaryotic cell lines
☒	☐ Palaeontology and archaeology
☒	☐ Animals and other organisms
☒	☐ Clinical data
☒	☐ Dual use research of concern

Methods

n/a	Involved in the study
☒	☐ ChIP-seq
☐	☒ Flow cytometry
☒	☐ MRI-based neuroimaging

Antibodies

Antibodies used

PerCP/Cy5.5 anti-human CD3 antibody, HIT3a clone, Biolegend, cat.300328;
APC-Cy7 anti-human CD4 antibody, OKT4 clone, Biolegend, cat. 317418;
Anti-CD3 monoclonal antibody, OKT3 clone, ThermoFisher, cat. 16-0037-81;
APC anti-human CD4, RPA-T4 clone, Biolegend, cat. 300514;
Pacific blue anti-human CD8, SK1 clone, Biolegend, cat. 344718;
APC anti-human CD45RA, HI100 clone, Biolegend, cat. 304112;
PE-Cy7 anti-human HLA-DR, L243 clone, Biolegend, cat. 307616;
CellTrace™ CFSE Cell Proliferation Kit, for flow cytometry, ThermoFisher, cat. C34554;
CD45RA MicroBeads, human, Miltenyi Biotec, cat. 130-045-901;
ELISA MAX™ Standard Set Human IFN-γ, Biolegend, cat. 430101;
LEGENDplex Human T Helper Cytokine Panels, Biolegend, cat. 740001;
LIVE/DEAD™ Fixable Yellow Dead Cell Stain Kit, for 405 nm excitation, ThermoFisher, cat. L34968;
LIVE/DEAD™ Fixable Aqua Dead Cell Stain Kit, for 405 nm excitation, ThermoFisher, cat. L34957;
Human GM-CSF DuoSet ELISA, rndsystems, cat. DY215;
Human XL Cytokine Luminex® Performance Assay 44-plex Fixed Panel, rndsystems,cat. LKTM014;
LIVE/DEAD Fixable Near-IR Dead Cell Stain Kit, ThermoFisher, cat. L10119;
Recombinant Anti-HLA-DR antibody, clone TAL 1B5, abcam, cat. ab20181;
CD68 Monoclonal Antibody, clone KP1, eBioscience, cat. 14-0688-82;
CD3ε, clone D7A6E, Cell Signaling technology, cat. 85061;
CD31/PECAM-1 Antibody, clone JC/70A, Novus Biologicals, cat. NB600-562;
Recombinant Anti-GFAP antibody, clone EP672Y, abcam, cat. ab220820;
Anti-Human IgG (Fc specific), Sigma-Aldrich, cat. I2136;
TCRBV1-PE, clone BL37.2, Beckman coulter, cat. IM2355;
TCRBV2-PE, clone MPB2D5, Beckman coulter, cat. IM2213;
TCRBV4-PE, clone WJF24, Beckman coulter, cat. IM3602;
TCRBV5.3-PE, clone 3D11, Beckman coulter, cat. IM2002;
TCRBV7.1-PE, clone ZOE, Beckman coulter, cat. IM2287;
TCRBV9-PE, clone FIN9, Beckman coulter, cat. IM2003;
TCRBV12-PE, clone VER2.32.1, Beckman coulter, cat. IM2291;
TCRBV13.1-PE, clone IMMU 222, Beckman coulter, cat. IM2292;
TCRBV14-PE, clone CAS1.1.3, Beckman coulter, cat. IM2047;
TCRBV18-PE, clone BA62.6, Beckman coulter, cat. IM2049;
TCRBV20-PE, clone ELL1.4, Beckman coulter, cat. IM2295;
TCRBV23-PE, clone AF23, Beckman coulter, cat. IM2004;
TCRBV3-FITC, clone CH92, Beckman coulter, cat. IM2372;
TCRBV5.1-FITC, clone IMMU 157, Beckman coulter, cat. IM1552;
TCRBV5.2-FITC, clone 36213, Beckman coulter, cat. IM1482;
TCRBV8-FITC, clone 56C5.2, Beckman coulter, cat. IM1233;
TCRBV11-FITC, clone C21, Beckman coulter, cat. IM1586;
TCRBV13.6-FITC, clone JU74.3, Beckman coulter, cat. IM1330;
TCRBV16-FITC, clone TAMAYA1.2, Beckman coulter, cat. IM1560;
TCRBV17-FITC, clone E17.5F3.15.13, Beckman coulter, cat. IM1234;
TCRBV21.3-FITC, clone IG125, Beckman coulter, cat. IM1483;
TCRBV22-FITC, clone IMMU 546, Beckman coulter, cat. IM1484;

Validation

All antibodies were validated by the manufacturer and data is available at the manufacturer's website as indicated below:
https://www.biolegend.com/it-it/products/percp-cyanine5-5-anti-human-cd3-antibody-5613
https://www.biolegend.com/fr-fr/products/apc-cyanine7-anti-human-cd4-antibody-3658
https://www.thermofisher.com/antibody/product/CD3-Antibody-clone-OKT3-Monoclonal/16-0037-81
https://www.biolegend.com/en-us/products/apc-anti-human-cd4-antibody-823?GroupID=BLG7755
https://www.biolegend.com/fr-ch/products/pacific-blue-anti-human-cd8-antibody-6509
https://www.biolegend.com/ja-jp/products/apc-anti-human-cd45ra-antibody-684
https://www.biolegend.com/nl-be/products/pe-cyanine7-anti-human-hla-dr-antibody-2862
https://www.thermofisher.com/order/catalog/product/C34554
https://www.miltenyibiotec.com/CH-en/products/cd45ra-microbeads-human.html#130-045-901
https://www.biolegend.com/fr-ch/products/human-ifn-gamma-elisa-max-standard-2226

https://www.biolegend.com/en-us/products/legendplex-human-th-cytokine-panel-13-plex-9699?GroupID=GROUP25
https://www.thermofisher.com/order/catalog/product/L34968
https://www.thermofisher.com/order/catalog/product/L34957
https://www.rndsystems.com/products/human-gm-csf-duoset-elisa_dy215
https://www.rndsystems.com/products/human-xl-cytokine-luminex-performance-assay-44-plex-fixed-panel_lktm014
https://www.thermofisher.com/order/catalog/product/L10119
https://www.abcam.com/hla-dr-antibody-tal-1b5-ab20181.html
https://www.thermofisher.com/antibody/product/CD68-Antibody-clone-KP1-Monoclonal/14-0688-82
https://www.cellsignal.com/products/primary-antibodies/cd3e-d7a6e-xp-rabbit-mab/85061
https://www.novusbio.com/products/cd31-pecam-1-antibody-jc-70a_nb600-562
https://www.abcam.com/gfap-antibody-ep672y-bsa-and-azide-free-ab220820.html
https://www.sigmaaldrich.com/CH/en/product/sigma/i2136
https://www.beckman.ch/reagents/coulter-flow-cytometry/antibodies-and-kits/single-color-antibodies/tcr-vb-3/im2372
https://www.beckman.ch/reagents/coulter-flow-cytometry/antibodies-and-kits/single-color-antibodies/tcr-vb-5-1/im1552
https://www.beckman.ch/reagents/coulter-flow-cytometry/antibodies-and-kits/single-color-antibodies/tcr-vb-5-2/im1482
https://www.beckman.ch/reagents/coulter-flow-cytometry/antibodies-and-kits/single-color-antibodies/tcr-vb-8/im1233
https://www.beckman.ch/reagents/coulter-flow-cytometry/antibodies-and-kits/single-color-antibodies/tcr-vb11/im1586
https://www.beckman.ch/reagents/coulter-flow-cytometry/antibodies-and-kits/single-color-antibodies/tcr-vb13-6/im1330
https://www.beckman.ch/reagents/coulter-flow-cytometry/antibodies-and-kits/single-color-antibodies/tcr-vb16/im1560
https://www.beckman.ch/reagents/coulter-flow-cytometry/antibodies-and-kits/single-color-antibodies/tcr-vb17/im1234
https://www.beckman.ch/reagents/coulter-flow-cytometry/antibodies-and-kits/single-color-antibodies/tcr-vb21-3/im1483
https://www.beckman.ch/reagents/coulter-flow-cytometry/antibodies-and-kits/single-color-antibodies/tcr-vb22/im1484
https://www.beckman.ch/reagents/coulter-flow-cytometry/antibodies-and-kits/single-color-antibodies/tcr-vb-1/im2355
https://www.beckman.ch/reagents/coulter-flow-cytometry/antibodies-and-kits/single-color-antibodies/tcr-vb-2/im2213
https://www.beckman.ch/reagents/coulter-flow-cytometry/antibodies-and-kits/single-color-antibodies/tcr-vb-4/im3602
https://www.beckman.ch/reagents/coulter-flow-cytometry/antibodies-and-kits/single-color-antibodies/tcr-vb-5-3/im2002
https://www.beckman.ch/reagents/coulter-flow-cytometry/antibodies-and-kits/single-color-antibodies/tcr-vb-7-1/im2287
https://www.beckman.ch/reagents/coulter-flow-cytometry/antibodies-and-kits/single-color-antibodies/tcr-vb-9/im2003
https://www.beckman.ch/reagents/coulter-flow-cytometry/antibodies-and-kits/single-color-antibodies/tcr-vb12/im2291
https://www.beckman.ch/reagents/coulter-flow-cytometry/antibodies-and-kits/single-color-antibodies/tcr-vb13-1/im2292
https://www.beckman.ch/reagents/coulter-flow-cytometry/antibodies-and-kits/single-color-antibodies/tcr-vb14/im2047
https://www.beckman.ch/reagents/coulter-flow-cytometry/antibodies-and-kits/single-color-antibodies/tcr-vb18/im2049
https://www.beckman.ch/reagents/coulter-flow-cytometry/antibodies-and-kits/single-color-antibodies/tcr-vb20/im2295
https://www.beckman.ch/reagents/coulter-flow-cytometry/antibodies-and-kits/single-color-antibodies/tcr-vb23/im2004

Eukaryotic cell lines

Policy information about cell lines and Sex and Gender in Research

Cell line source(s)

BLS cells expressing HLA-DRA1*01:01 and BLS-DRB1*03:01 were kindly gifted from William W. Kwok, Benaroya institute BLS-DRB1*04:02, BLS-DRB3*02:02 and BLS-DRB4*01:01 were created in-house. All BLS cell lines in this study were originally derived from a female.

Authentication

Gifted cell lines were authenticated by the suppliers.
BLS cells were subjected to antibiotic selection in culture to deplete cells that have lost their HLA-DR expression. BLS cells expressing different HLA-DRB molecules were then authenticated using HLA-DR antibody for expression of HLA-II molecules. All BLS cells were expressing HLA-DR molecule. No further authentication has been conducted.

Mycoplasma contamination

All cells were regularly tested for mycoplasma contamination and were negative.

Commonly misidentified lines
(See ICLAC register)

No commonly misidentified lines were used in this study

Flow Cytometry

Plots

Confirm that:

☒ The axis labels state the marker and fluorochrome used (e.g. CD4-FITC).

☒ The axis scales are clearly visible. Include numbers along axes only for bottom left plot of group (a 'group' is an analysis of identical markers).

☒ All plots are contour plots with outliers or pseudocolor plots.

☒ A numerical value for number of cells or percentage (with statistics) is provided.

Methodology

Sample preparation

CFSE-labeled bulk T cells were seeded with irradiated PBMCs loaded with peptides. After incubation, cells were washed and stained with live-dead dye and human IgG for 30 minutes. Next, cells were washed and stained with the detection antibodies for 30 minutes in 4 degree. Finally, cells were washed and used for FACS or sorting.

Instrument	LSR Fortessa Flow Cytometer (BD Biosciences) was used to measure cytokines and sorting was conducted using a SH800S Cell Sorter (Sony).
Software	Data were analyzed using FlowJo (Tree Star).
Cell population abundance	Known cell numbers based on the counted and seeded cells in each well were used for TILs, CD45RA- PBMCs and T cell clones
Gating strategy	Cells were always gated for live, proliferating (CFSEdim) and further gated based on the staining

☒ Tick this box to confirm that a figure exemplifying the gating strategy is provided in the Supplementary Information.
